# A sequence-based global map of regulatory activity for deciphering human genetics

**DOI:** 10.1038/s41588-022-01102-2

**Published:** 2022-07-11

**Authors:** Kathleen M. Chen, Aaron K. Wong, Olga G. Troyanskaya, Jian Zhou

**Affiliations:** 1 Department of Computer Science, Princeton University, Princeton, NJ USA; 2 Flatiron Institute, Simons Foundation, New York, NY USA; 3 Lewis-Sigler Institute for Integrative Genomics, Princeton University, Princeton, NJ USA; 4 Lyda Hill Department of Bioinformatics, University of Texas Southwestern Medical Center, Dallas, TX USA

**Keywords:** Computational biology and bioinformatics, Genetics, Genomics

## Abstract

Epigenomic profiling has enabled large-scale identification of regulatory elements, yet we still lack a systematic mapping from any sequence or variant to regulatory activities. We address this challenge with Sei, a framework for integrating human genetics data with sequence information to discover the regulatory basis of traits and diseases. Sei learns a vocabulary of regulatory activities, called sequence classes, using a deep learning model that predicts 21,907 chromatin profiles across >1,300 cell lines and tissues. Sequence classes provide a global classification and quantification of sequence and variant effects based on diverse regulatory activities, such as cell type-specific enhancer functions. These predictions are supported by tissue-specific expression, expression quantitative trait loci and evolutionary constraint data. Furthermore, sequence classes enable characterization of the tissue-specific, regulatory architecture of complex traits and generate mechanistic hypotheses for individual regulatory pathogenic mutations. We provide Sei as a resource to elucidate the regulatory basis of human health and disease.

## Abstract (web summary)

Sei is a new framework for integrating human genetics data with a sequence-based mapping of predicted regulatory activities to elucidate mechanisms contributing to complex traits and diseases.

## Main

Deciphering how regulatory functions are encoded in genomic sequences is a major challenge in understanding how genome variation links to phenotypic traits. Cell type-specific regulatory activities encoded in elements such as promoters, enhancers and boundary elements are critical to defining the complex expression programs essential for multicellular organisms. Most disease-associated variants from genome-wide association studies (GWAS) are located in noncoding regions^1^, yet without knowing how changes in sequence affect regulatory activities, we cannot predict the impact of these variants and uncover the regulatory mechanisms contributing to complex diseases and traits.

Substantial progress has been made in the experimental profiling and integrative analysis of epigenomic marks, such as histone marks and DNA accessibility, across a wide range of tissues and cell types^2–4^. At the same time, deep learning sequence modeling techniques have been successfully applied to learn sequence features predictive of transcription factor (TF) binding and histone modifications^5–11^. These models are powerful tools for inferring the impact of sequence variation at the chromatin level—for example, whether a variant increases or decreases C/EBPβ binding. However, we continue to lack an integrative view of sequence regulatory activities, including all major aspects of *cis*-regulatory functions, such as tissue-specific or broad enhancer and promoter activities. This limits our ability to interpret the integrated effects of all chromatin-level perturbations caused by genomic variants and determine their impact on human health and diseases.

We address this challenge by creating a global map for sequence regulatory activity based on a new deep learning-based framework called Sei. This framework introduces a sequence model that predicts 21,907 publicly available chromatin profiles—the broadest set to date—and uses the model to quantitatively characterize regulatory activities for any sequence with a vocabulary we call sequence classes. Sequence classes cover diverse types of regulatory activities, such as promoter or cell type-specific enhancer activity, across the whole genome by integrating sequence-based predictions from histone marks, TFs and chromatin accessibility across a wide range of cell types. Importantly, sequence classes can be used to both classify and quantify the regulatory activities of any sequence based on predictions made by the deep learning sequence model, thereby allowing any mutation to be quantified by its impact (for example, increase, decrease or no change) on cell type-specific regulatory activities.

Thus, the Sei framework enables an interpretable and systematic integration of sequence-based regulatory activity predictions with human genetics data to elucidate the regulatory basis of complex traits and diseases. We applied our framework to characterize disease- and trait-associated regulatory disruptions in GWAS data based on a nonoverlapping partitioning of heritability by regulatory activities. Moreover, we applied variant effect prediction at the sequence class-level to interpret cell type-specific regulatory mechanisms for individual disease mutations and differentiate between gain-of-function (GoF) and loss-of-function (LoF) regulatory mutations.

We provide the Sei framework as a resource for systematically classifying and scoring any sequence and variant with sequence classes, additionally providing the Sei model predictions for the 21,907 chromatin profiles underlying the sequence classes. The framework can be run using the code available at https://github.com/FunctionLab/sei-framework; a user-friendly web server is available at hb.flatironinstitute.org/sei.

## Results

### Developing a comprehensive, chromatin-level sequence model

To capture the widest range of sequence features predictive of regulatory activities, we first developed a new deep learning sequence model, which we refer to as the Sei model that enables the base-level interpretation of sequences by predicting 21,907 genome-wide *cis*-regulatory targets—including peak calls from 9,471 TF binding, 10,064 histone mark and 2,372 chromatin accessibility profiles—with single-nucleotide sensitivity. Most of this data (19,905 profiles) was processed by the Cistrome Project^4^; the remaining chromatin profiles were processed by the ENCODE^2^ and Roadmap Epigenomics^3^ projects. The Sei model encompasses an estimated 1,000 nonhistone DNA-binding proteins (which we refer to as TFs), 77 histone marks and chromatin accessibility across >1,300 cell lines and tissues (Supplementary Tables 1 and 2).

To efficiently predict 21,907 chromatin profiles from sequence, we designed a new residual block architecture with dual linear and nonlinear paths, which takes as input a 4-kilobase (kb) length sequence and predicts the probabilities of 21,907 *cis*-regulatory targets at the center position (Supplementary Fig. 1 and Methods).

The model achieved an average area under the receiver operating characteristic (AUROC) of 0.972 and average area under the precision-recall curve (AUPRC) of 0.409 across all 21,907 chromatin profiles (Supplementary Fig. 2). In addition to accurately predicting individual profiles, Sei predictions recapitulated their correlation structure, indicating that the model captures the colocalization patterns of chromatin profiles (Supplementary Fig. 3). Furthermore, the Sei model improved over our best previously published model, DeepSEA Beluga^7^, on the 2,002 chromatin profiles predicted by both models by 19% on average (measured by AUROC/(1 − AUROC); Supplementary Fig. 4).

Therefore, the Sei model is the most comprehensive chromatin-level sequence model to date and offers an expansive new resource for sequence and variant interpretation.

### Defining sequence classes from sequence model predictions

Next, we applied the Sei model to develop a global, quantitative map from genomic sequences to specific classes of regulatory activities, which we termed sequence classes, by integrating the wide range of chromatin profiles predicted by Sei. Therefore, sequence classes were mapped directly from sequence, with each class representing a distinct program of regulatory activities across the tissues and cell types covered by the Sei model. Furthermore, sequence classes allow for the mapping of any sequence to quantitative scores that represent a broad spectrum of regulatory activities.

**Fig. 1:**
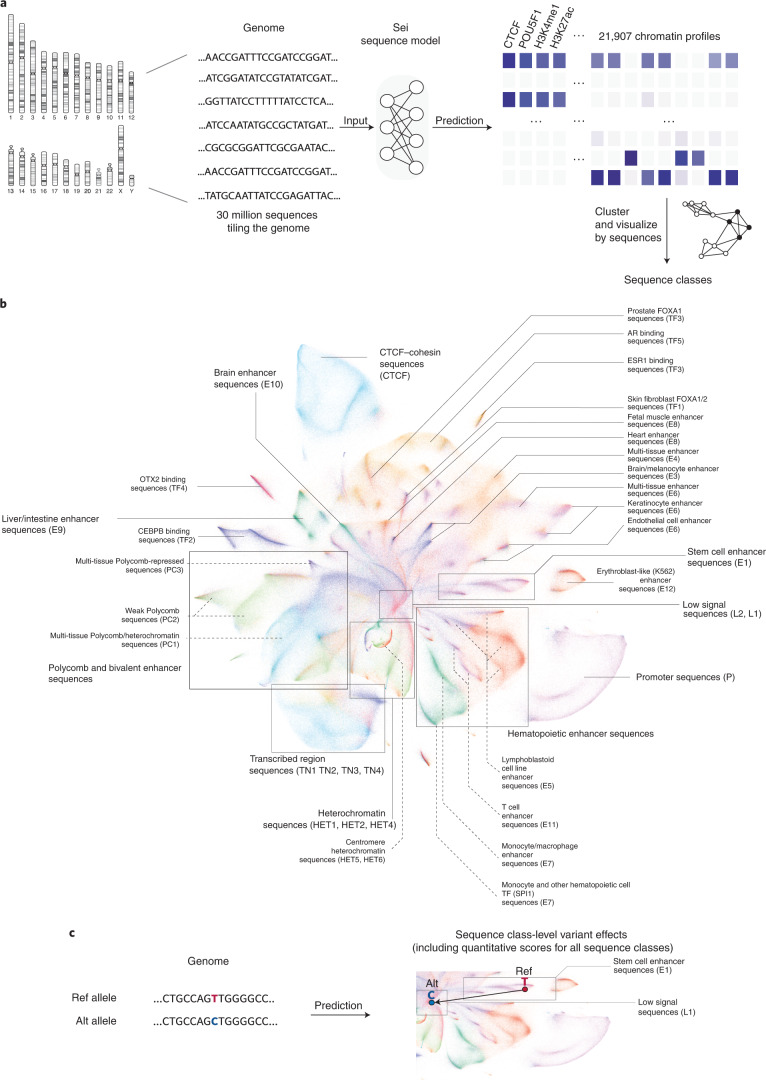
Mapping the global regulatory landscape of genomic sequences. **a**, Overview of the Sei framework for systematic prediction of sequence regulatory activities. Sequence classes were extracted from the predicted chromatin profiles of 30 million sequences evenly tiling the genome. The predictions were made by Sei, a new deep convolutional network sequence model trained on 21,907 chromatin profiles. Specifically, classes are identified by applying Louvain community detection to the nearest neighbor graph of 180 principal components extracted from the predictions data. **b**, Visualizing the global regulatory landscape of human genome sequences discovered by this approach with UMAP. Major sequence classes include cell type-specific enhancer classes, CTCF–cohesin, promoter, TF-specific and heterochromatin/centromere classes. AR, androgen receptor. **c**, This framework was further applied to predict sequence class-level genome variant effects, quantified by changes in sequence class scores.

Here we identified sequence classes from Sei predictions for 30 million sequences uniformly tiling the whole genome (4-kb windows with 100-base pair (bp) step size) by applying Louvain community clustering^12^ to these predictions to categorize the 30 million sequences into 40 sequence classes (Fig. 1a). We then visualized the global structure of sequence regulatory signals as represented by the model’s chromatin profile predictions using nonlinear dimensionality reduction techniques^13,14^ (Fig. 1).

This visualization of human genome sequences demonstrates the global organization of sequence regulatory activities (Fig. 1b). The center of the visualization contains sequences with weak or no regulatory activity based on histone mark and TF enrichment—sequences with specific regulatory activities radiate outward from there. Different branches of sequences are enriched in distinct chromatin modifications and TFs, and sequences with similar regulatory activities are grouped together. For example, tissue-specific enhancer sequences were predominantly grouped by tissue in the visualization (Fig. 1b). In addition, sequences with repressive Polycomb marks were spatially adjacent to H3K9me3-marked heterochromatin sequences (Fig. 1b), reflecting their extensive crosstalk in epigenetic silencing^15–17^. Promoter-proximal and CTCF–cohesin binding sequences form two well-defined clusters separate from other sequences, which may indicate the distinct nature of these activities (Fig. 1b).

**Fig. 2:**
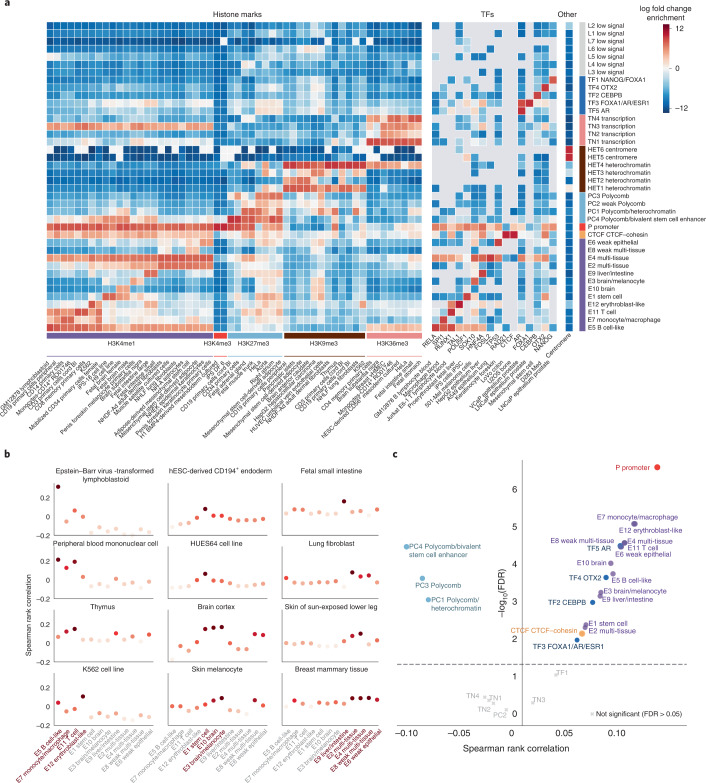
Sequence classes predict cell type-specific regulatory activities and directional, expression-altering variant effects. **a**, Sequence class-specific enrichment of histone marks, TFs and repeat annotations; log fold change enrichment over genome-average background is shown in the heatmap. No overlap is indicated by the gray color in the heatmap. The top 1–2 histone mark and TF annotation enrichments were selected for each sequence class. hESC, human embryonic stem cell. **b**, Enhancer sequence classes near TSS were correlated with cell type-specific gene expression in the applicable tissue or cell types (Methods). The *y* axis shows the Spearman rank correlation between the proportion of each sequence class annotation within 10 kb of TSS and the tissue-specific differential gene expression (fold over tissue average). **c**, Regulatory sequence class-level variant effects are predictive of directional GTEx variant gene expression effects. The *x* axis shows Spearman correlations between the predicted sequence-class-level variant effects and the signed GTEx variant effect sizes (slopes) for variants with strong predicted effects near the TSS (Methods); the *y* axis shows the corresponding −log_10_ *P* values. All colored dots are above the Benjamini–Hochberg false discovery rate (FDR) <0.05 threshold.

The sequence classes identified from whole-genome sequences provide a basis for summarizing sequence activities globally. They recapitulate the sequence organization shown in the visualization (Fig. 1b) and are robust to changes in clustering parameter choices (Supplementary Figs. 5 and 6). Each sequence class is labeled with a functional group acronym and index denoting the rank of the class within the group (Supplementary Fig. 13; for example, E1 encompasses a larger proportion of the genome than E2). Because genomic sequences encode their regulatory activity programs across all cell types, sequence classes show distinct activity patterns across cell types and tissues (Fig. 2a, Supplementary Figs. 7–12 and Supplementary Data). To facilitate intuitive interpretation of sequence classes, they are named primarily by their active, cell type-specific regulatory activities—in particular, enrichment of promoter and enhancer activities. In other words, sequence classes not labeled as enhancer (E) or promoter (P) generally lack enhancer or promoter activity in any cell type predicted by Sei.

Based on their enrichment in relevant histone marks and TFs, sequence classes consist of the following categories: P, promoter; E, enhancer; CTCF; TF; PC, Polycomb; HET, heterochromatin; TN, transcription; and L, low signal, which are not strongly enriched in the measured histone marks (Fig. 2a, Supplementary Figs. 7–12 and Supplementary Data). For example, the P promoter class is enriched in the active promoter histone mark H3K4me3 across all cell types (Fig. 2a and Supplementary Fig. 7). The 12 E enhancer classes are strongly enriched in enhancer histone marks (H3K4me1, H3K27ac) and TFs relevant to their activities in select cell types (for example, PU.1/SPI1 in E7 monocytes/macrophages, SOX2/NANOG/POU5F1 in E1 stem cells) and often display repressive H3K27me3 marks in inactive cell types (Fig. 2a, Supplementary Figs. 8–10 and Supplementary Data). As a whole, the 40 sequence classes cover >97.4% of the genome (Supplementary Fig. 13).

Beyond classification of genomic sequences, sequence classes provide a global and quantitative scoring of sequence regulatory activities. This for the first time allows us to (1) predict the regulatory activity for any sequence and (2) quantify the changes in regulatory activity caused by any variant (Fig. 1c). Sequence class scores summarize predictions for all 21,907 chromatin profiles based on weights specific to each sequence class. Scores are computed by projecting Sei predictions onto unit-length vectors that point to the center of each sequence class; the impact of a variant is represented by the difference between scores for the reference (Ref) and alternative (Alt) alleles. Sequences that score highly for a particular sequence class have high predictions for the chromatin profiles associated with that class. While sequence classes defined using Sei model predictions are highly concordant with those defined from chromatin profiling data (Supplementary Fig. 14), defining sequence classes based on Sei model predictions enables the mapping of any sequence to sequence classes beyond the reference genome. Importantly, this capability cannot be directly obtained from chromatin profiling data alone.

### Enhancer sequence classes predict tissue-specific expression

The group of sequences that likely have the most impact on tissue-specific gene expression regulation are the enhancer (E) sequence classes; thus, we assessed the association of enhancer sequence class scores with tissue-specific gene expression.

In the visualization of sequence regulatory activities, sequence classes with different cell type- and tissue-specific enhancer activities are localized to distinct subregions (Fig. 1b). E sequence classes capture both specific and broad enhancer activities (Figs. 1b and 2a). For example, based on enhancer mark enrichment, E7 is specific for monocytes and macrophages, E9 is specific for liver and intestine, E1 is specific for embryonic and induced pluripotent stem cells (iPSCs) and E10 and E3 are specific for brain (Fig. 2a and Supplementary Figs. 8 and 9; all enrichments stated are significant with *P* < 2.2 × 10^−16^, two-sided Fisher’s exact test). In contrast, broad enhancer sequence classes such as E2 and E4 encompass enhancer activity in many different cell types (for example, fibroblast, muscle, osteoblast, epithelial). Sequence class enhancer activities are also supported by the enrichment of relevant chromatin states^2^ and DNase I hypersensitive sites (DHSs)^18^ across tissues and cell types (Supplementary Figs. 15 and 16). Consistent with their predicted cell type-specific activities, the coverage of E sequence class annotations within a 10-kb window to transcription start sites (TSS) are correlated with the differential expression patterns of these genes in the corresponding cell types over the tissue average (Fig. 2b).

Since sequence class scores allow us to systematically predict the effects of variants on regulatory functions, we can estimate whether a given variant diminishes, maintains or increases the enhancer activity of a sequence. Evaluated on Genotype-Tissue Expression (GTEx) expression quantitative trait loci (eQTL) data^19^, we found that variants predicted to increase E sequence class activity were significantly positively correlated with higher gene expression, whereas those predicted to increase PC sequence class activity were significantly negatively correlated with gene expression, consistent with the expected repressive role of PC sequence class activities (Fig. 2c). Moreover, when only analyzing fine-mapped eQTLs^20^ with high posterior inclusion probability (>0.95), we observed higher correlations with overall comparable levels of significance (Supplementary Fig. 17). Therefore, sequence classes can distinguish the effects of variants on gene expression based on their consequences in regulatory activities.

### Sequence classes are under evolutionary constraints

**Fig. 3:**
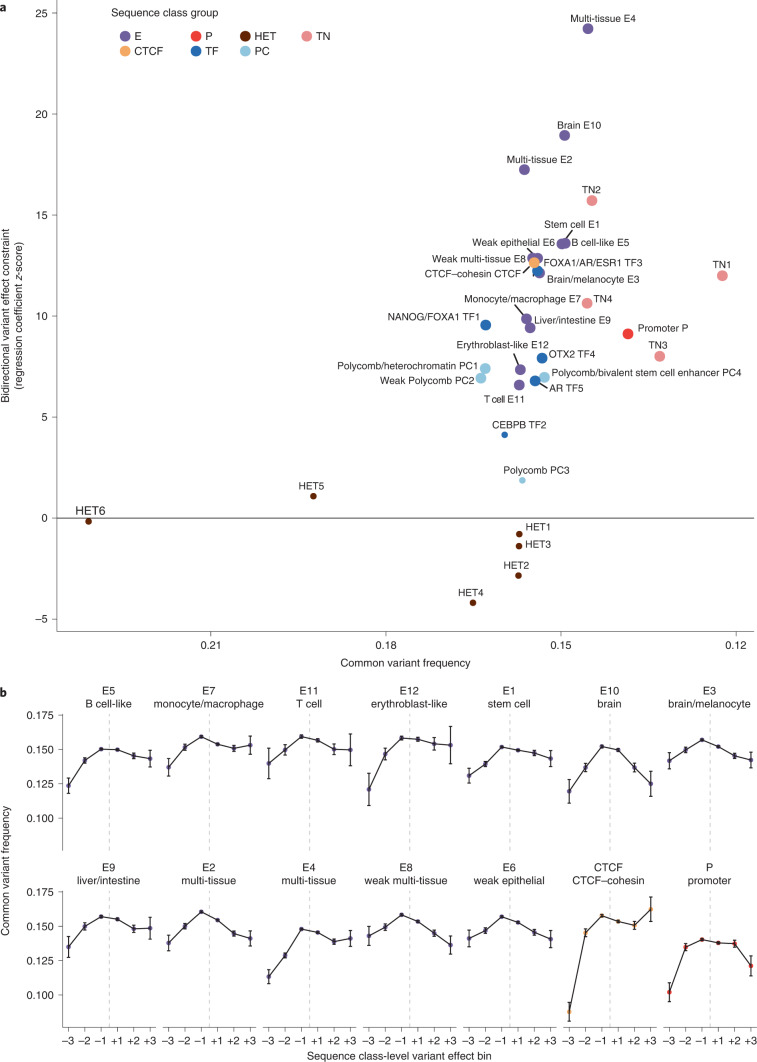
Variants with strong regulatory sequence class effects show negative selection signatures. **a**, Scatter plot for AF-based analysis of each sequence class. The *x* axis shows 1 − common variant frequency (AF >0.01) across all 1000 Genomes variants per sequence class; the *y* axis shows the bidirectional variant effect constraint *z*-score, which was computed based on logistic regressions predicting a common variant (AF >0.01) from the sequence class-level variant effect score for both positive and negative effects (Methods). Sequence classes with significant (Benjamini–Hochberg FDR <0.05) bidirectional variant effect constraint are indicated with larger dots. L sequence classes are excluded due to lack of interpretation for their sequence class-level variant effect scores. **b**, Comparison of common variant frequencies for 1000 Genomes variants (*n* = 81,501,608) assigned to different sequence classes and variant effect bins. The common variant threshold is >0.01 AF across the 1000 Genomes population (*n* = 12,803,919). The error bars show ±1 s.e. and the center of the error bars represents the mean. The sequence class-level variant effects are assigned to six bins (+3, top 1% positive; +2, top 1–10% positive; +1, top 10–100% positive; −1, top 10–100% negative; −2, top 1–10% negative; −3, top 1% negative).

Variants that alter the regulatory activities of sequences often disrupt gene regulation and are therefore expected to impact human health and disease. We tested this expectation by comparing human population genome variant allele frequencies (AFs)^21^ based on the sequence class in which each variant is located and the predicted variant effect on that class. Indeed, we found that variants localized in regulatory sequence classes (E, P and CTCF) have lower common variant frequency than variants in other sequence classes and showed higher overall negative selection constraint (Fig. 3a, *x* axis). More importantly, variants predicted to strongly perturb regulatory sequence classes are significantly less likely to be common variants than variants that weakly perturb these classes (measured by bidirectional variant effect constraint; Fig. 3a, *y* axis and Fig. 3b and Methods). Therefore, this is consistent with the hypothesis that disruption of regulatory sequence class activities has a major negative impact on fitness, which we refer to as a negative selection signature.

Specifically, we observed strong negative selection signatures for variants assigned to all E, CTCF and P sequence classes (Fig. 3). Multi-tissue enhancer sequence classes E4 and E2 and the brain enhancer sequence class E10 showed the strongest association of the predicted sequence class-level variant effect and the probability of a variant being a common variant. Notably, for the CTCF sequence class, only negative variant effects—decreasing sequence class activity—appear to be under very strong constraints, suggesting that CTCF sites are generally tolerant to positive effect mutations that further increase CTCF binding. This is in contrast to the generally deleterious impact of both increase and decrease of enhancer and promoter activities. As expected, TN sequence classes, which overlap with protein-coding regions, are among the sequence classes with the lowest AF (Supplementary Fig. 18).

Variants assigned to the HET, PC, TF and L sequence classes generally did not show strong negative selection signatures (Supplementary Fig. 18). Importantly, this does not suggest that PC or TFs are inessential: PC-related regulation is likely critical for E and P sequence classes, which are often PC-repressed in some cell types but enhancers or promoters in other cell types (Supplementary Figs. 7–10). Similarly, we expect that TF binding plays a central role in E classes that are highly enriched in relevant TFs (Fig. 2a and Supplementary Data).

Therefore, sequence classes show distinct evolutionary constraints and E enhancer sequence classes show the strongest bidirectional constraints. This suggests that both increases and decreases of enhancer activity are expected to lead to deleterious effects on fitness, highlighting the importance of precisely controlling gene expression.

### The regulatory architecture of GWAS traits

The population AF analysis on sequence classes suggests that variants perturbing regulatory sequence class activities are likely to be involved in human health and disease. To explore this hypothesis, we used GWAS data to delineate the genetic contribution of each sequence class to diseases and traits.

**Fig. 4:**
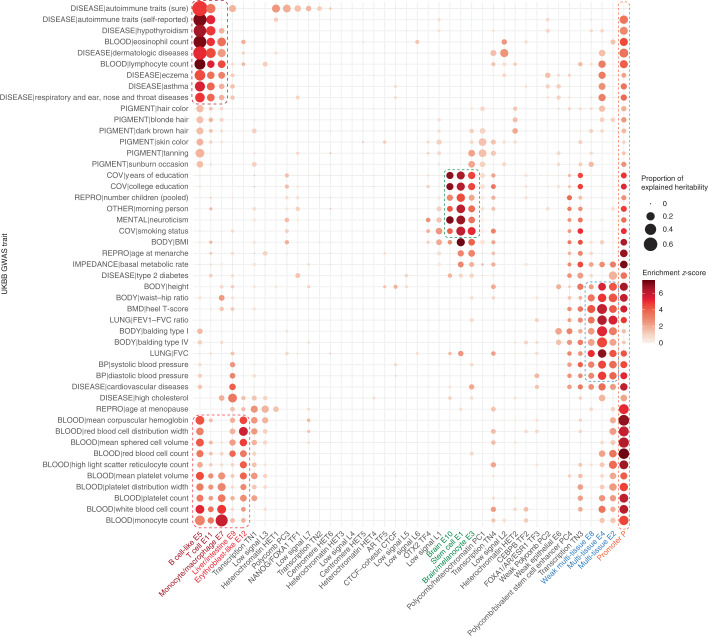
Sequence class-based partitioning of GWAS heritability shows trait associations with tissue-specific regulation. Partitioned genome-wide heritability in the UKBB GWAS with all 40 sequence classes. The size of the dot indicates the proportion of heritability estimated from LDSR, which is conservatively estimated as 1 s.e. below the estimated heritability proportion (bounded by 0). The color of the dot indicates the significance *z*-score of the fold enrichment of heritability relative to the proportion of all SNPs assigned to the sequence class (bounded by 0). The colored boxes indicate traits associated with blood (red), brain (green), multiple tissues (blue) and promoters (orange). BMI, body mass index; FEV1, forced expiratory volume in one second.

Partitioned heritability from linkage disequilibrium (LD) score regression (LDSR) has been a powerful tool for understanding the genetic architecture of diseases and traits using GWAS summary statistics^22^, including identifying enrichment of disease heritability in regulatory elements^22,23^. Previous applications of LDSR used overlapping annotations^22–24^, which allow for the joint analysis of heritability contribution across annotations and have generated important insights into a wide range of traits; however, such analyses cannot unambiguously partition heritability into nonoverlapping categories. Because sequence classes are both nonoverlapping (that is, each variant is assigned to one sequence class) and cover nearly the entire genome, they provide a clear and easily interpretable picture of the regulatory architecture of diseases and traits. To show this, we estimated the proportion of heritability explained by each sequence class for 47 GWAS traits in the UK Biobank (UKBB)^25,26^ (Methods). Our analysis of the UKBB GWAS revealed genetic signatures of sequence class-specific regulatory functions (Fig. 4 and Supplementary Table 3).

Importantly, E and P sequence classes, which are also inferred to be under strong evolutionary constraints, cover almost all classes that explain a high proportion of heritability for GWAS traits and diseases (Fig. 3a and Supplementary Table 3). We observed three main groups of traits that share similar heritability composition signatures across sequence classes. The first group encompasses blood-related traits and contains two subgroups of immune- and nonimmune-related traits. Most of the heritability signals in blood-related traits are explained by enhancer classes for the relevant cell type(s), such as monocyte/macrophage enhancer (E7) for ‘monocyte count’ (Fig. 4). Furthermore, autoimmune-related traits are selectively associated with the immune cell-related enhancer sequence classes E5 (B cell-like), E11 (T cell) and E7 (monocyte/macrophage), while red blood cell-related traits are linked to erythroblast-like enhancer E12. Therefore, sequence classes can dissect the cell type-specific regulatory architecture of traits and diseases with heritability decomposition, even without relying on gene-level information.

Cognitive and behavioral traits (‘morning person’, ‘neuroticism’, ‘smoking status’, ‘years of education’, ‘college education’) also share similar sequence class-level heritability decompositions; in this group, heritability was mostly explained by brain (E10 and E3) and stem cell (E1) enhancer sequence classes. The link to E1 is consistent with our observation that E1 was also moderately enriched for the active enhancer mark H3K4me1 in brain cell types (Fig. 2a and Supplementary Fig. 7) and is positively correlated with gene expression in brain tissues (Fig. 2b).

The third group of traits is intriguingly diverse, including ‘balding’, ‘lung forced vital capacity (FVC)’, ‘waist–hip ratio’, ‘height’ and ‘heel T-score’. The heritability of these traits is mostly explained by multi-tissue enhancer classes (E4, E2 and E8), which show activity in epithelial cells, fibroblasts, muscle and many other cell types. Enhancer activity across multiple tissues in the body may explain the diverse phenotypes that are associated with these traits.

Beyond these three groups, there are a number of traits with unique heritability patterns that are also linked to highly relevant sequence classes. For example, the ‘high cholesterol’ trait was most associated with the liver and intestine enhancer sequence class (E9), which is consistent with the physiology of cholesterol metabolism and known etiology of this condition^27^. E9 was also linked to red blood cell-related traits, in line with the role of liver in erythropoiesis.

Finally, the promoter sequence class P uniquely explained a sizable proportion of heritability in nearly all traits, suggesting a near-universal involvement of promoter sequence variations in all traits and diseases.

We next assessed whether sequence classes could explain GWAS heritability beyond that explained by annotations discovered in previous studies. We uncovered 83 significant sequence class-trait associations after conditioning on published baseline annotations (Supplementary Table 4 and Methods). We found that 33 out of 47 of all UKBB GWAS traits and 9 out of 13 of the E and P sequence classes have at least 1 significant association after multiple hypothesis testing correction (Supplementary Table 4). This finding suggests that sequence classes can identify extensive new regulatory signals that enrich GWAS interpretation.

### Disease mutations disrupt sequence class activities

**Fig. 5:**
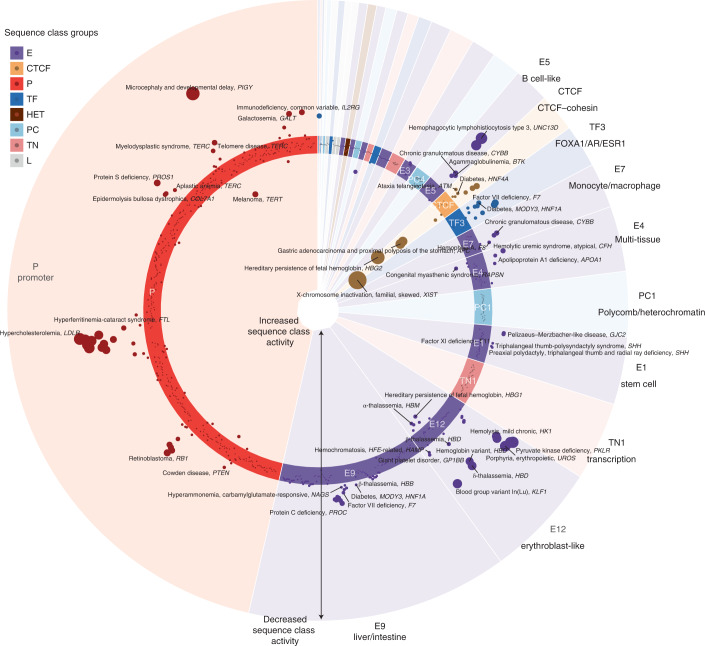
Disease regulatory mutations are predicted to disrupt promoter, CTCF and tissue-specific enhancer sequence classes. Sequence class-level mutation effects of pathogenic noncoding HGMD mutations were plotted. A polar coordinate system was used, where the radial coordinate indicates the sequence class-level effects. Each dot represents a mutation and mutations inside the circle are predicted to have positive effects (increased activity of sequence class); mutations outside the circle are predicted to have negative effects (decreased activity of sequence class). Dot size indicates the absolute value of the effect. Mutations were assigned to sequence classes based on their sequences and predicted effects (Methods). Within each sequence class, mutations were ordered by chromosomal coordinates. The associated disease and gene name were annotated for each mutation and only the strongest mutation was annotated if there were multiple mutations associated with the same disease, gene and sequence class.

Sequence class-level effects enable the prediction of specific regulatory mechanisms at the individual, pathogenic mutation level. To showcase our framework’s capability to predict the mechanisms of individual mutations, we used Sei to predict the direction and magnitude of sequence class-level mutation effects for all 853 regulatory disease mutations from the Human Gene Mutation Database (HGMD)^28^ (Fig. 5). For systematic classification and quantification of these mutations, we assigned each mutation to an affected sequence class (Methods).

Overall, the average variant effect score of disease mutations is 4.2 times larger than that of de novo mutations in healthy individuals (0.903 versus 0.217, *P* <2.2 × 10^−16^, two-sided Wilcoxon rank-sum test, maximum absolute effect across sequence classes) and 6.5 times larger than the 1000 Genomes common variants with AF >0.01 (0.903 versus 0.139, *P* < 2.2 × 10^−16^). In this study, we focused on analyzing mutations with the strongest predicted effects (>1.1, *n* = 138 out of 853) (Fig. 5 and Supplementary Fig. 19). Because sequence class-level variant effects are directional—that is, predicting whether the Alt allele increases or decreases sequence class-level activity—we discovered that while most (approximately 80%) of pathogenic mutations with strong predicted effects are predicted to decrease sequence class activity, the remaining 20% of HGMD pathogenic mutations are predicted to increase sequence class activity. Moreover, perturbations to the E, P and CTCF classes make up >99% of the mutations with strong predicted effects on sequence class activity (Supplementary Table 5), with 44.9% predicted to affect tissue-specific E sequence classes, 38.4% predicted to affect the P sequence class and, interestingly, 15.9% predicted to affect the CTCF–cohesin sequence class (Methods).

Almost all mutations with strong predicted effects in cell type-specific E sequence classes contributed to diseases relevant to that same cell type (Fig. 5 and Supplementary Table 5). For most of these mutations, the nearby gene is known to be relevant to the disease but the molecular mechanisms of regulatory disruption is unknown. For example, mutations causing vitamin K-dependent protein C deficiency and hemophilia B, two diseases characterized by deficiency of specific plasma proteins produced in the liver, are predicted to decrease E9 liver/intestine sequence class activities. Blood cell-specific enhancer sequence classes (for example, E12 erythroblast-like, E7 monocyte/macrophage-like and E5 B cell-like) are disrupted in distinct blood-related diseases and deficiencies relevant to the corresponding cell type. For developmental diseases, such as preaxial triphalangeal thumb-polysyndactyly syndrome, the E1 embryonic stem cell-specific enhancer sequence class is predicted to be disrupted by mutations in a known distal enhancer of sonic hedgehog signaling molecule (*SHH*) (chromosome 7: 156,583,949 G>C)^29^, a gene that plays a crucial role in the positioning and growth of limbs, fingers and toes during development.

In addition, 38% of the regulatory mutations with strong predicted effects disrupt P sequence class activity (Fig. 5). The high proportion of mutations perturbing the P class likely reflects both the critical role of promoters in diseases and the emphasis on promoter-proximal mutations in past studies.

While the mutations we have discussed thus far are negative effect mutations (decreasing sequence class activity), 20% of HGMD pathogenic mutations are predicted to increase sequence class activity. Indeed, these mutations include many known GoF mutations, which validate our predictions. The highest increase in sequence class activity was observed for a mutation (X chromosome: 73,072,592 G>C) near the *XIST* gene that skews X-inactivation of the mutant chromosome in females^30^; this mutation was predicted to increase the activity of the CTCF sequence class and has been experimentally validated to increase CTCF binding^31^. Similarly, positive effect predictions for E and P sequence classes were validated by previously studied mutations: an α-thalassemia mutation near *HBM* (chromosome 16: 209,709 T>C)^32^ known to create a GATA1 binding site and increase intergenic transcription was predicted to increase erythroblast-specific E12 activity; a *TERT* mutation found in individuals with familial melanoma (chromosome 5: 1,295,161 T>G)^33^ was predicted to increase P activity. Beyond this, many mutations predicted to have strong positive effects were not previously understood. For example, a mutation near the *HBG1* gene (chromosome 11: 5,271,262 A>G)^34^ causing persistence of fetal hemoglobin is predicted to increase erythroblast-specific E12 sequence class activity. Previously, this mutation was known to create an ATGCAAAT octamer^34^ that matches the POU family TF motif but its functional consequences were unclear.

Taken together, sequence class-level effects both corroborate existing regulatory mechanisms and propose new mechanisms for individual pathogenic mutations. We expect our framework to be a valuable tool in accelerating genetic discoveries of disease causal mutations and their mechanisms in the regulatory genome.

## Discussion

We developed a genome-wide sequence-based map of regulatory activities using sequence classes, a vocabulary for genomic sequence activities discovered using a data-driven, systematic method. Our deep learning-based framework uses a compendium covering 21,907 publicly available *cis*-regulatory profiles and the whole-genome sequence to create a mapping from any sequence to a comprehensive set of sequence classes. Sequence classes are a concise vocabulary of regulatory activities that is interpretable, quantifiable and easily analyzed globally (across all sequence classes) and individually.

We demonstrated that E and P sequence classes are strongly enriched in trait and disease GWAS heritability and under evolutionary constraints. Importantly, sequence classes provide insights into the mechanisms of individual pathogenic mutations by predicting effects on the function of tissue-specific enhancers, promoter activity and long-range genome interactions (for example, the CTCF–cohesin sequence class). Using sequence class-level variant effect predictions, we linked many pathogenic mutations to tissue-specific regulatory changes in the relevant tissues. These predictions point to potential mechanisms that can be tested experimentally in the future.

This work demonstrates the potential of sequence classes to discover regulatory disruptions in human diseases, through both the aggregation of genome-wide variant association signals and the prediction of the impact of individual mutations. We expect sequence classes and the Sei model to be a powerful tool for understanding the mechanistic effects of noncoding mutations in human health.

## Methods

### Training data

A total of 21,907 *cis*-regulatory profiles in peak format were compiled from the processed files of the Cistrome^4^, ENCODE^2^ and Roadmap Epigenomics projects^3^. The Cistrome Project, which systematically processes publicly available *cis*-regulatory profiles, contributed most of the profiles predicted in Sei (*n* = 19,905). We excluded profiles from Cistrome with fewer than 1,000 peaks. Genome sequences are from the GRCh38/hg38 human reference genome. The full list of *cis*-regulatory profiles is available in Supplementary Table 1.

### Deep learning sequence model training

The Sei model was trained to predict 21,907 TF binding, histone marks and DNA accessibility from *cis*-regulatory profile peaks at the center of 4-kb length sequences. The model is trained on chromatin profile peak calls, which are binary (presence/absence), but the model output is continuous, representing probabilities of peaks.

The model architecture is composed of three sequential sections: (1) a convolutional network with dual linear and nonlinear paths; (2) residual dilated convolution layers; (3) spatial basis function transformation and output layers. A detailed specification of the model is available in Supplementary Fig. 21 and in the code repository (https://github.com/FunctionLab/sei-framework, downloadable from 10.5281/zenodo.4906996). In the convolutional architecture, we introduced a new design composed of both linear and nonlinear convolution blocks. The linear path allows for fast and statistically efficient training, while the nonlinear path offers strong representation power and the capability to learn complex interactions. The nonlinear blocks consist of convolution layers and rectified linear activation functions, similar to regular convolutional networks. The linear blocks have the same structure as the nonlinear blocks but do not include activation functions to facilitate learning of linear dependencies. Each nonlinear block is stacked on top of a linear block with a residual connection adding the input of the nonlinear block to the output, allowing the computation to go through either the linear or nonlinear path. Dilated convolutional layers with residual connections further expand the receptive fields without reducing spatial resolution. Finally, for scaling and performance, we introduced a layer of spatial basis functions, which integrates information across spatial locations with much higher memory efficiency than fully connected layers. Spatial basis functions are used to reduce dimensionality of the spatial dimension while preserving the capability to discriminate spatial patterns of sequence representations. Specifically, in the Sei model, a B-spline basis matrix (256 × 16) with 16 degrees of freedom across 256 uniformly spaced spatial bins is generated and multiplied with the convolutional layers output to reduce the 256 spatial dimensions to 16 spline basis function dimensions. After the spline basis function transformation, a fully connected layer and an output layer are used to integrate information across the whole sequence and generate the final 21,907 dimensional predictions.

Our model training pipeline was updated^35^ to improve training speed and performance by using on-the-fly sampling, which reduces overfitting by generating new training samples for every training step. Training, validation and testing datasets are specified by different sets of chromosomes in the hg38 genome (holding out chromosome 8 and 9 for the test set and chromosome 10 for the validation set) and samples drawn uniformly across the hg38 genome for these partitions, excluding regions specified in the ENCODE blacklist^36^. For training, we sampled training sequences and their labels on the fly from the training set of chromosomes using Selene^35^. Thus almost all training samples are drawn from unique genomic intervals with distinct start and end positions to reduce overfitting during the training process. For each 4-kb region, a 21,907 dimensional binary label vector was created for the 21,907 *cis*-regulatory profiles based on whether the center bp overlaps with a peak in each of the profiles. The model was implemented in PyTorch and trained with Selene. A detailed training configuration file is available at https://github.com/FunctionLab/sei-framework/blob/main/train/train.yml.

### Model performance

We computed the AUROC and AUPRC for all *cis*-regulatory profiles predicted by Sei on the test holdout dataset, excluding profiles that had fewer than 25 positive samples in the test set. Additionally, to assess the correlation structure of the predictions, we compared the rank-transformed pairwise Spearman rank correlations for the predicted *cis*-regulatory profiles to the pairwise correlations for the true labels (peak calls provided in the Cistrome Data Browser).

The model performance comparison between DeepSEA and Sei was computed on the 2,002 *cis*-regulatory profiles from Roadmap and ENCODE that both DeepSEA and Sei predict. Because both models have the same chromosomal test holdout (chromosomes 8 and 9), we use the regions specified in the DeepSEA test holdout set to create a common test dataset of sequences and labels on which to evaluate the models.

### Sequence classes

We selected 30 million genomic positions that uniformly tile the genome with a 100-bp step size and then computed Sei predictions for 4-kb sequences centered at each position. Sequences overlapping with ENCODE blacklist regions^36^ or assembly gaps (Ns) were removed. To process the 30 million × 21,907 predictions matrix, the dimensionality was first reduced with principal component analysis (PCA). The PCA transformations were fitted with incremental PCA using a batch size of 1,000,000 for 1 pass of the whole dataset; genomic positions were randomly assigned to batches. The top 180 principal components, scaled to unit variance, were used for constructing a nearest neighbor graph where each node is connected to its *k*-nearest neighbors by Euclidean distance (*k* = 14). Louvain community clustering with default parameters was applied to the nearest neighbor graph with the python-louvain package (version 0.6.1), which resulted in 61 clusters. We refer to the largest 40 clusters as sequence classes and exclude the remaining (smallest) 21 clusters, which constitute <2.6% of the genome, from our analyses due to their size. These 21 clusters mainly displayed L or HET-like enrichment (Supplementary Fig. 20). We refer to this cluster assignment to sequence classes at 100-bp resolution as sequence class annotations. We visualized the genome-wide predictions by computing uniform manifold approximation and projection (UMAP) embedding with a subsample of PCA-transformed Sei predictions of 30 million sequences and then fine-tuned the visualization with openTSNE (version 0.6.0). The detailed procedures are available in our code repository (https://github.com/FunctionLab/sei-manuscript).

### Sequence class scores

Each sequence class is represented as a unit vector in the 21,907 dimensional *cis*-regulatory profile space, in the direction of the average prediction of all sequences assigned to this sequence class among the 30 million. In more formal notation, the vector for sequence class *i* is $v_i = \frac{{\overline {p_{s \in {{{\mathrm{Sequence}}}}\,{{{\mathrm{class}}}}\,i}} }}{{\overline {||p_{s \in {{{\mathrm{Sequence}}}}\,{{{\mathrm{class}}}}\,i}||_2} }}$, where *p*_*s*_ represents the 21,907 dimensional Sei prediction for sequence *s*. Each Sei prediction can then be projected onto any sequence class vector to obtain a sequence class-level representation of the prediction, which we call sequence class score or ${{{\mathrm{score}}}}_{s,\,i} = p_s^T \cdot v_i$. In addition, predicted sequence class-level variant effects are represented by the difference between the sequence class scores of the sequences carrying the Ref and Alt alleles or ${{{\mathrm{score}}}}_{v,\,i} = {{{\mathrm{score}}}}_{{{{\mathrm{Alt}}}},\,i} - {{{\mathrm{score}}}}_{{{{\mathrm{Ref}}}},\,i}$. To better represent predicted variant effects on histone marks, it is necessary to normalize for nucleosome occupancy (for example, a LoF mutation near the TSS can decrease H3K4me3 modification level while increasing nucleosome occupancy, resulting in an overall increase in observed H3K4me3 quantity). Therefore, for variant effect computation, we used the sum of all histone profile predictions as an approximation to nucleosome occupancy and adjusted all histone mark predictions to remove the impact of nucleosome occupancy change (nonhistone mark predictions are unchanged):

$$p_{{{{\mathrm{Ref}}}}}^{{{{\mathrm{hm}}^\ast}}} = p_{{{{\mathrm{Ref}}}}}^{{{{\mathrm{hm}}}}}\frac{{\mathop {\sum}\nolimits_k {p_{{{{\mathrm{Ref}}}}}^{{{{\mathrm{hm}}}}^k}} + \mathop {\sum}\nolimits_k {p_{{{{\mathrm{Alt}}}}}^{{{{\mathrm{hm}}}}^k}} }}{{2\mathop {\sum}\nolimits_k {p_{{{{\mathrm{Ref}}}}}^{{{{\mathrm{hm}}}}^k}} }};\,p_{{{{\mathrm{Alt}}}}}^{{{{\mathrm{hm}}^\ast}} } = p_{{{{\mathrm{Alt}}}}}^{{{{\mathrm{hm}}}}}\frac{{\mathop {\sum}\nolimits_k {p_{{{{\mathrm{Ref}}}}}^{{{{\mathrm{hm}}}}^k}} + \mathop {\sum}\nolimits_k {p_{{{{\mathrm{Alt}}}}}^{{{{\mathrm{hm}}}}^k}} }}{{2\mathop {\sum}\nolimits_k {p_{{{{\mathrm{Alt}}}}}^{{{{\mathrm{hm}}}}^k}} }}$$

where $\mathop {\sum}\limits_k {p_{{{{\mathrm{Ref}}}}}^{{{{\mathrm{hm}}}}^k}}$ represents the sum over all histone mark predictions (among 21,907 dimensions of a prediction) for the Ref allele. We generally excluded L sequence classes in sequence class-level variant effect analyses because they lack an intuitive biological interpretation.

### Sequence class enrichment of chromatin profiles and genome annotations

We computed the log fold change enrichment of various chromatin profiles and genome annotations for each sequence class based on sequence class annotations (described above); log fold change enrichment is computed by taking the log ratio of the proportion of a sequence class intersecting with the annotation versus the background proportion of the annotation, where we consider all regions assigned to any sequence class. We computed enrichment for all 21,907 profiles predicted by Sei, filtered the chromatin profiles for each sequence class to only those having Benjamini–Hochberg-corrected (two-sided Fisher’s exact test) *P* < 2.2 × 10^−16^ and selected the top 25 profiles based on log fold change enrichment. Cistrome Project profile enrichment is computed over 2 million random genomic positions.

The annotation of centromere repeats was obtained from the University of California, Santa Cruz RepeatMasker track and annotations of histone marks over multiple cell types were obtained from the Roadmap Epigenomics project—enrichments for both of these sets of annotations were computed over the entire genome. In addition, we obtained ChromHMM chromatin states from ENCODE^2^ and tissue- and cell type-specific DHS vocabulary from Meuleman et al.^18^.

### Enhancer sequence class correlations with cell type-specific gene expression

Tissue expression profiles are from GTEx^19^, Roadmap Epigenomics^3^ and ENCODE^2^ and transformed to log RPKM (reads per kilobase of transcript, per million mapped reads) scores as previously described^7^ and normalized by tissue average. Specifically, a pseudocount was added before log transformation (0.0001 for GTEx tissues, which are averaged across individuals, and 0.01 for Roadmap and ENCODE tissues). After log transformation, the average scores across tissues were subtracted for each gene; thus, the processed scores represent log fold change relative to tissue average.

Gene-wide expression prediction was evaluated on sequence class annotations (from Louvain community clustering) for positions within ±10 kb of the TSS for these genes. For each enhancer sequence class and tissue, we computed a Spearman correlation between the sequence class annotation coverage and gene expression.

### Sequence class variant effect correlation with directional eQTL variant effect sizes

We collected the eQTLs within ±5 kb of gene TSS from GTEx v8, combined across all GTEx tissues, and computed the Spearman correlation between the top 15,000 variant effect predictions for each sequence class and the eQTL variant effect sizes (averaged across multiple tissues if the variant was an eQTL in multiple tissues). *P* values were derived from a two-sided Spearman rank correlation test and Benjamini–Hochberg correction was applied. L and HET sequence classes were excluded from this analysis due to lack of interpretation for their variant effect scores in this context.

Additionally, we collected fine-mapped GTEx eQTLs from the eQTL Catalogue^20^ and obtained sequence class scores for eQTLs with a posterior inclusion probability greater than 0.95. Variants were assigned to sequence classes based on the sequence class annotation for the reference genome (that is, variants were not further selected based on variant effect predictions). For each sequence class, we computed the Spearman rank correlation between sequence class scores and eQTL variant effect sizes in the same way we described above.

### Evolutionary constraints on variant effects

We computed sequence class-level variant effects for all 1000 Genomes Project phase 3 variants^21^. Variants were assigned to sequence classes based on the 100-bp resolution genome-wide assignment derived from Louvain community clustering. For each sequence class, we divided variants into six bins based on their effects in the same sequence class as illustrated in Fig. 3 and summarized the common variant (AF > 0.01) frequencies in each bin by mean and s.e.m. We also estimated statistical significance of AF dependency on sequence class-level variant effects. For each sequence class, we applied logistic regression separately for positive effect and negative effect variants to predict common variants (AF > 0.01) from the absolute value of the sequence class-level variant effect score and obtained the significance *z*-score of the regression coefficient of variant effect. The bidirectional evolutionary constraint *z*-score is defined as the negative value of the combined *z*-scores from positive and negative effect variants with Stouffer’s method.

### Partitioning GWAS heritability by sequence classes

The UKBB GWAS summary statistics were obtained from Loh et al.^25^. To study the association of sequence class genome annotation and sequence class variant effects and trait heritability, we performed partitioned heritability LDSR as described by Finucane at al.^22^. To partition the heritability as sums of heritability explained by each sequence class, we ran LDSR with only sequence class annotations and a baseline all-ones annotation. We obtained the estimated proportion of *h*^2^ explained by each sequence class and its s.e. with LDSR as implemented in https://github.com/bulik/ldsc. Because the estimated proportions can have high variance or even be negative (the true value of heritability explained can only be nonnegative), we used a robust and conservative estimator that is the estimated proportion of *h*^2^ subtracted by one s.e. and then lower-bounded by zero. (The s.e. of the estimated proportion of *h*^2^ explained is given by LDSR and was estimated with the block jackknife procedure as described by Finucane et al.^22^.)

To assess the contribution of sequence classes to explaining additional heritability when conditioned on known baseline annotations, we also ran LDSCORE v.2.2 with the baseline annotations (https://alkesgroup.broadinstitute.org/LDSCORE/). *P* values were derived from the coefficient *z*-score; Benjamini–Hochberg correction was applied.

### Sequence class variant effect analysis of noncoding pathogenic mutations

We obtained all mutations assigned ‘DM’ and ‘regulatory’ annotation in the HGMD database (2019.1 release). *RMRP* gene mutations were excluded because they are likely pathogenic due to impacting RNA function instead of regulatory perturbations, despite being annotated to the regulatory category in HGMD. For every mutation, we predicted the sequence class scores for both the Ref and Alt alleles and computed the sequence class-level variant effect as the predicted scores for the Alt allele subtracting the scores for the Ref allele. To provide an overview of sequence class-level effects of human noncoding pathogenic mutations, mutations were first assigned to sequence classes based on the sequence class annotations of the mutation position. For mutations with a strong effect in a different sequence class than the originally assigned sequence class (absolute value higher than the original sequence class by >1 absolute difference and >2.5-fold relative difference), we reassigned the mutation to the sequence class with the strongest effects.

### Reporting summary

Further information on research design is available in the Nature Research Reporting Summary linked to this article.

## Online content

Any methods, additional references, Nature Research reporting summaries, source data, extended data, supplementary information, acknowledgements, peer review information; details of author contributions and competing interests; and statements of data and code availability are available at 10.1038/s41588-022-01102-2.

## Supplementary information

Supplementary Information Supplementary Figs. 1–21.

Reporting Summary

Supplementary Tables Supplementary Tables 1–5.

Supplementary Data Top 25 enriched Cistrome Project chromatin profiles for each sequence class.

## Data Availability

The eQTL data were obtained from GTEx v.8 (ref. ^19^). Human population variants were obtained from the 1000 Genomes Project phase 3 (ref. ^21^). Human regulatory disease mutations were obtained from the HGMD (2019.1 release). All data for the manuscript results are available at https://github.com/FunctionLab/sei-manuscript.

## Code availability

The Sei framework code is available at https://github.com/FunctionLab/sei-framework and the model and associated data files can be downloaded by following the instructions in the GitHub repository. The code for the manuscript results is available at https://github.com/FunctionLab/sei-manuscript. Both repositories are available for download at 10.5281/zenodo.6502732.
