# Supplementary material for: A sequence-based global map of regulatory activity for deciphering human genetics
Source: Nat Genet. 2022 Jul 11;54(7):940–9. doi: 10.1038/s41588-022-01102-2 (PMC9279145; doi:10.1038/s41588-022-01102-2)
Supplement: Supplementary file 1 — Supplementary Figs. 1–21. [file 41588_2022_1102_MOESM1_ESM.pdf]

nature portfolio

https://doi.org/10.1038/s41588-022-01102-2

Supplementary information

A sequence-based global map of regulatory activity for deciphering human genetics

In the format provided by the authors and unedited

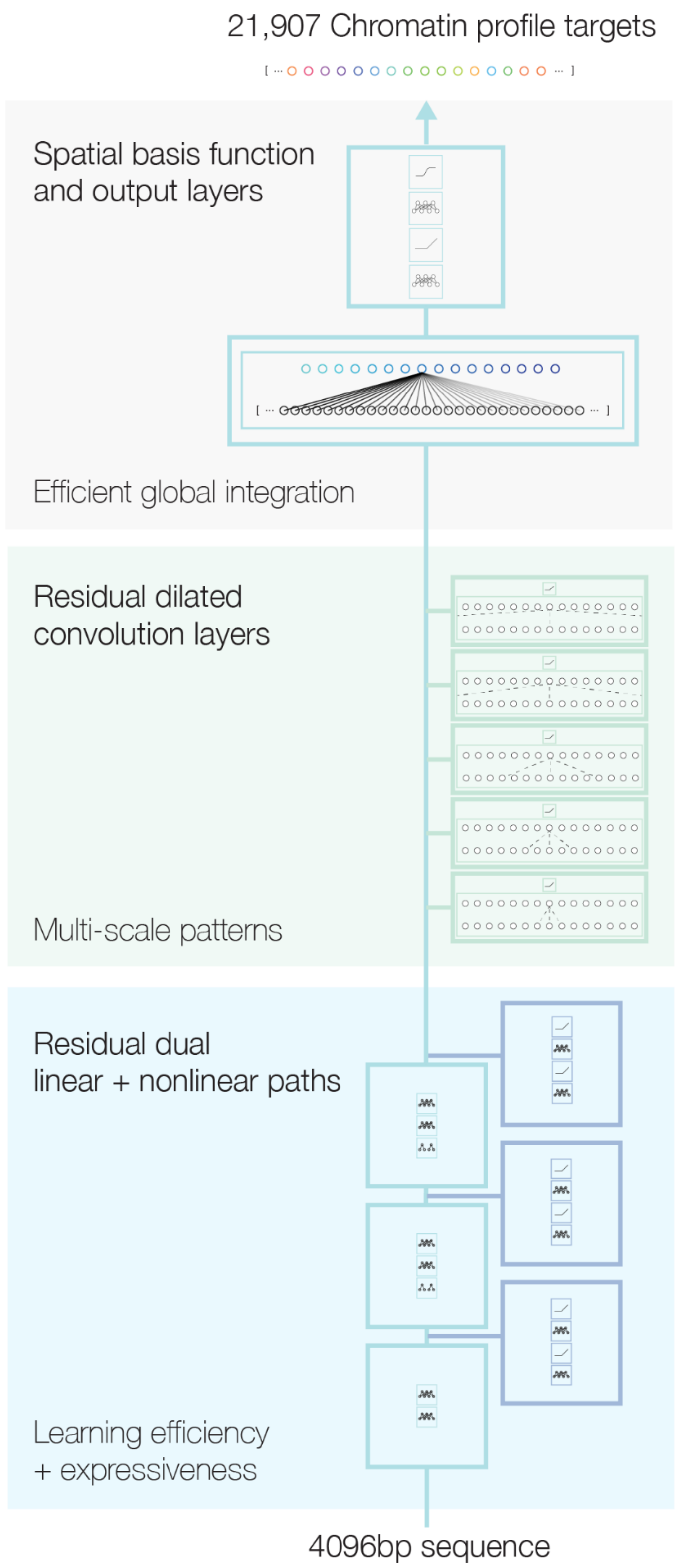

Supplementary Figure 1. Schematic overview of Sei model architecture. 4096bp sequences, one-hot encoded, are the input to the model (bottom) and the predicted 21,907 cis-regulatory profiles are the output (top).

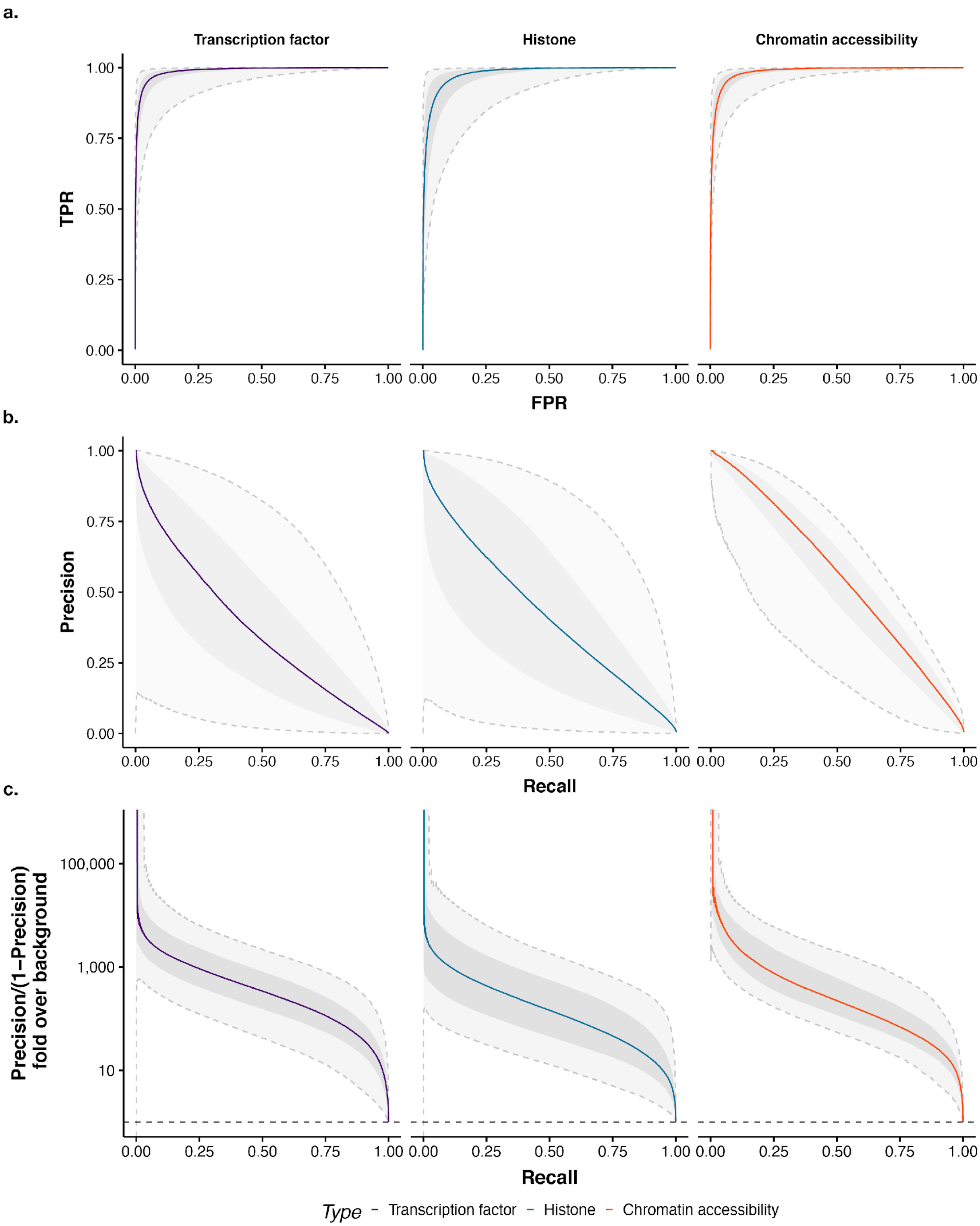

Supplementary Figure 2. Sei model performance on predicting 21907 cis-regulatory profiles on holdout chromosomes. a, AUROC curves; b, precision-recall curves; c, precision-recall curves normalized by the proportion of positives. For all plots, the median score is represented by the colored

line. The darker shaded region represents the 25th and 75th percentiles. The lighter shaded region (dashed lines) represents the 10th and 90th percentiles.

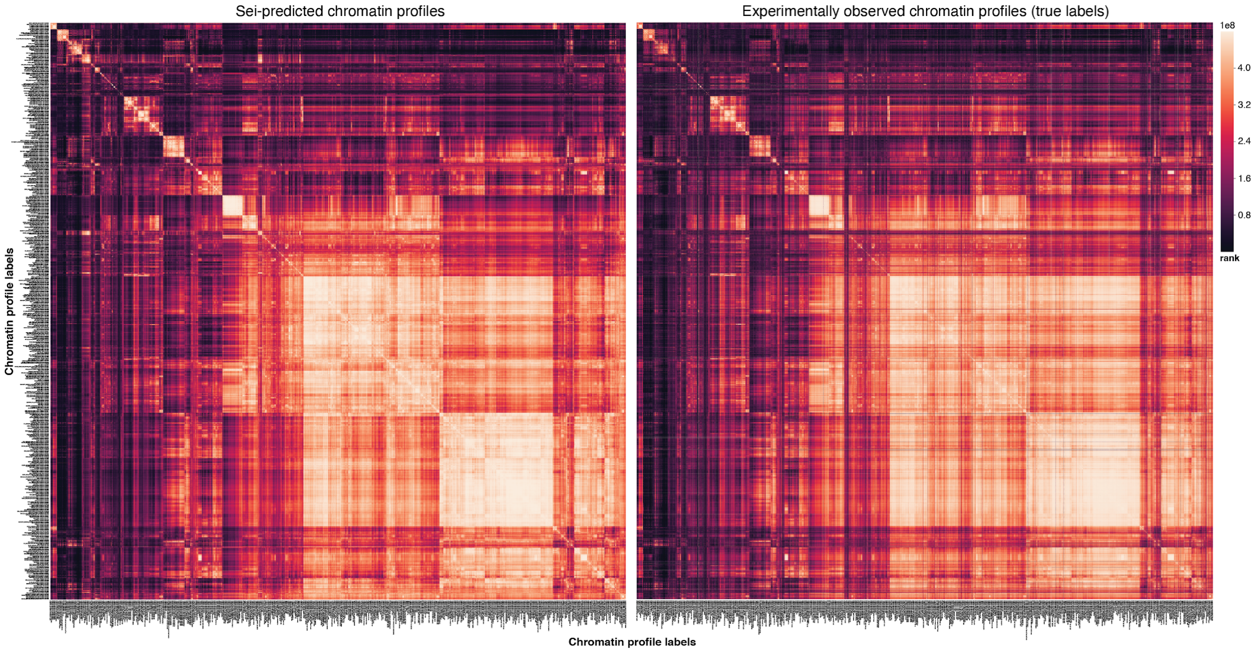

Supplementary Figure 3. Visualizing the rank-transform of pairwise Spearman correlations for the 21,907 cis-regulatory profiles in Sei. Sei model predictions share a highly similar correlation structure with the experimental observations.

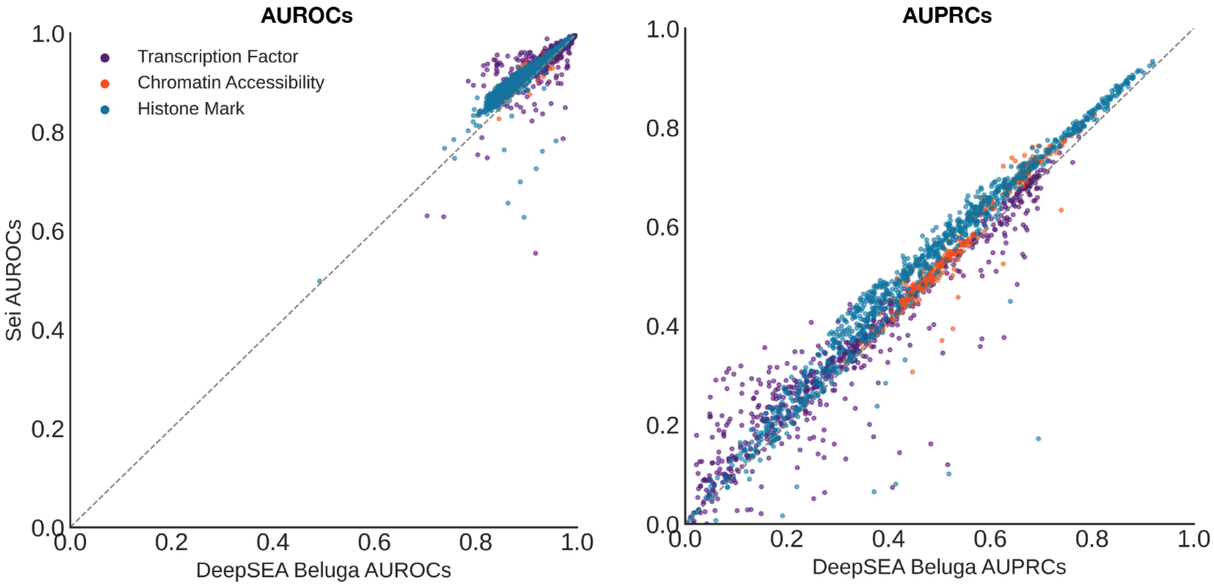

Supplementary Figure 4. Sei model performance comparison with DeepSEA. Performance on the shared 2002 DeepSEA "Beluga" (2018) cis-regulatory profiles are compared.

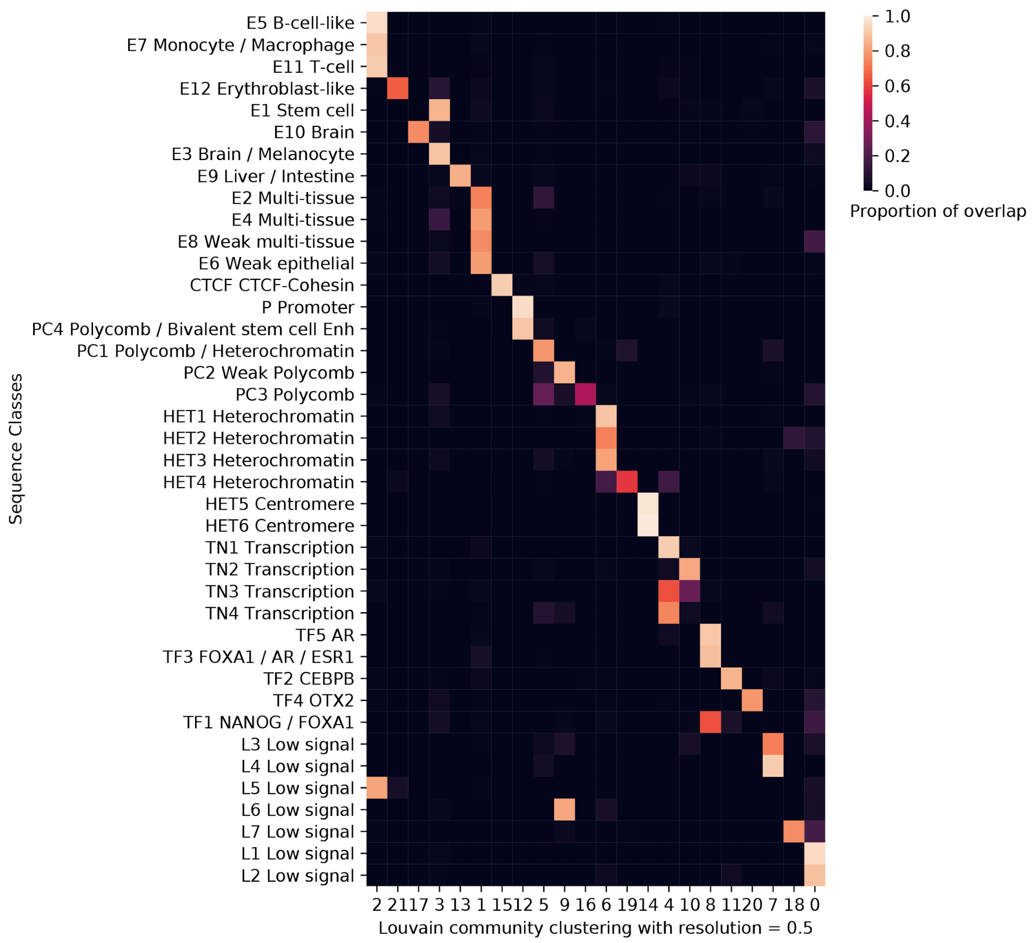

Supplementary Figure 5. Comparison of sequence classes and Louvain community clustering with resolution = 0.5. For each sequence class, the proportion of overlap was computed between sequence classes and a lower resolution clustering. The lower resolution clustering is largely consistent with the original sequence classes, with some clusters combining several related enhancer sequence classes into one.

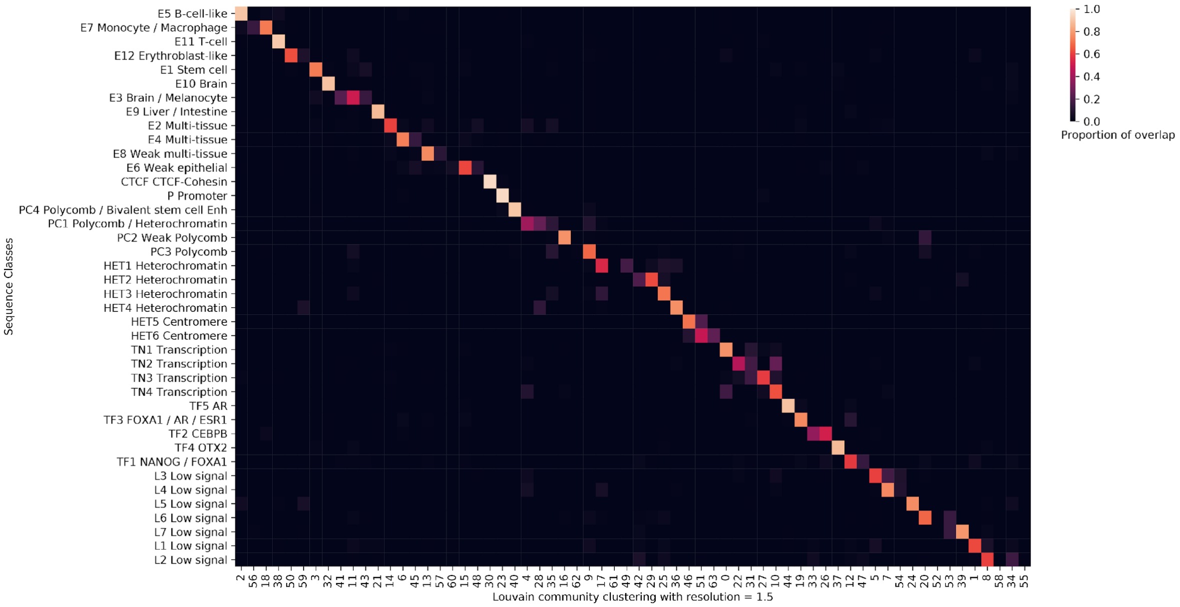

Supplementary Figure 6. Comparison of sequence classes and Louvain community clustering with resolution = 1.5. For each sequence class, the proportion of overlap was computed between sequence classes and a higher resolution clustering. The higher resolution clustering closely resembles the current sequence class clusters.

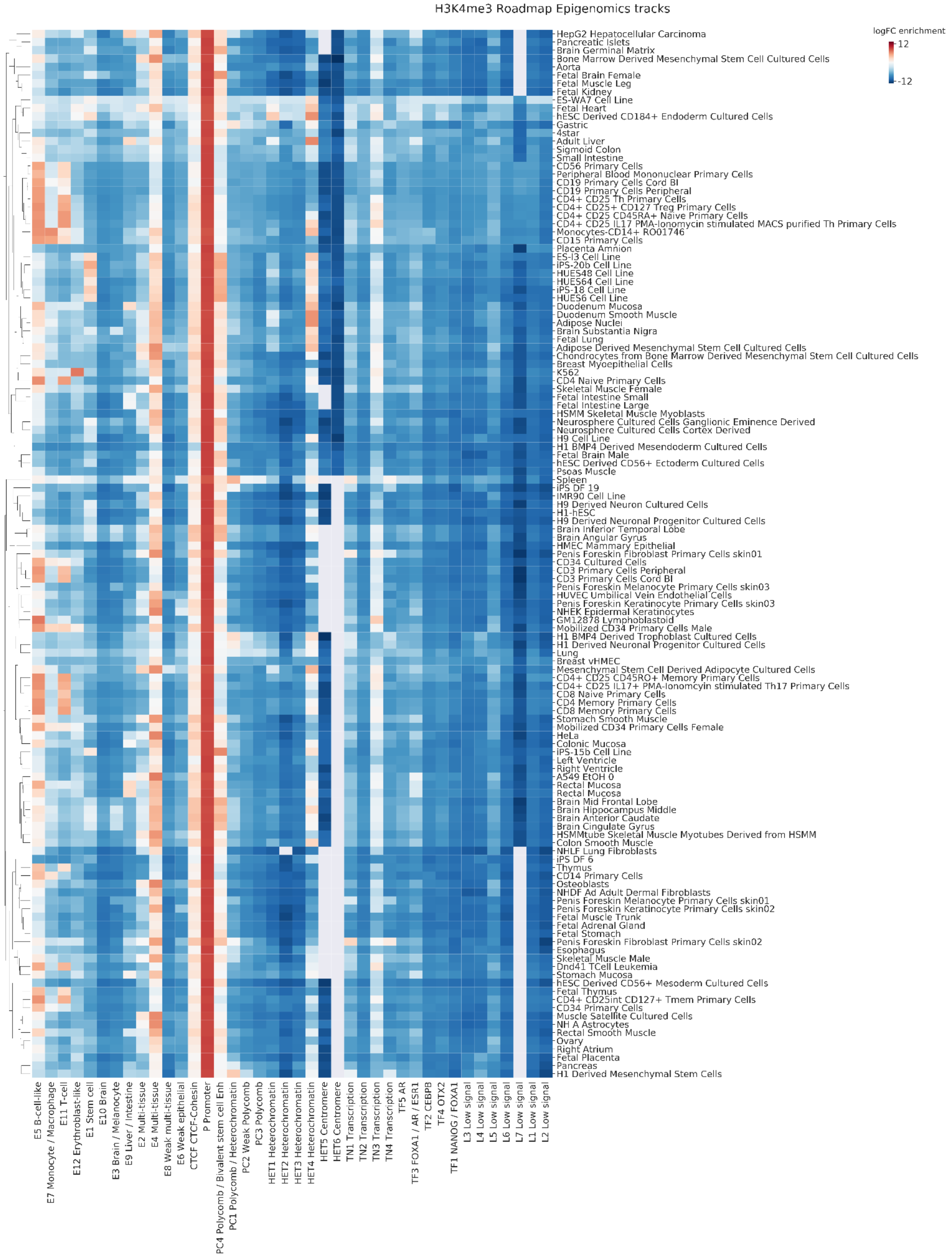

Supplementary Figure 7. Enrichment of tissue/cell type-specific H3K4me3 (promoter mark) profiles in sequence classes. Log fold change enrichment over genome-average background is shown in the heatmap. No overlap is indicated by the gray color in the heatmap.

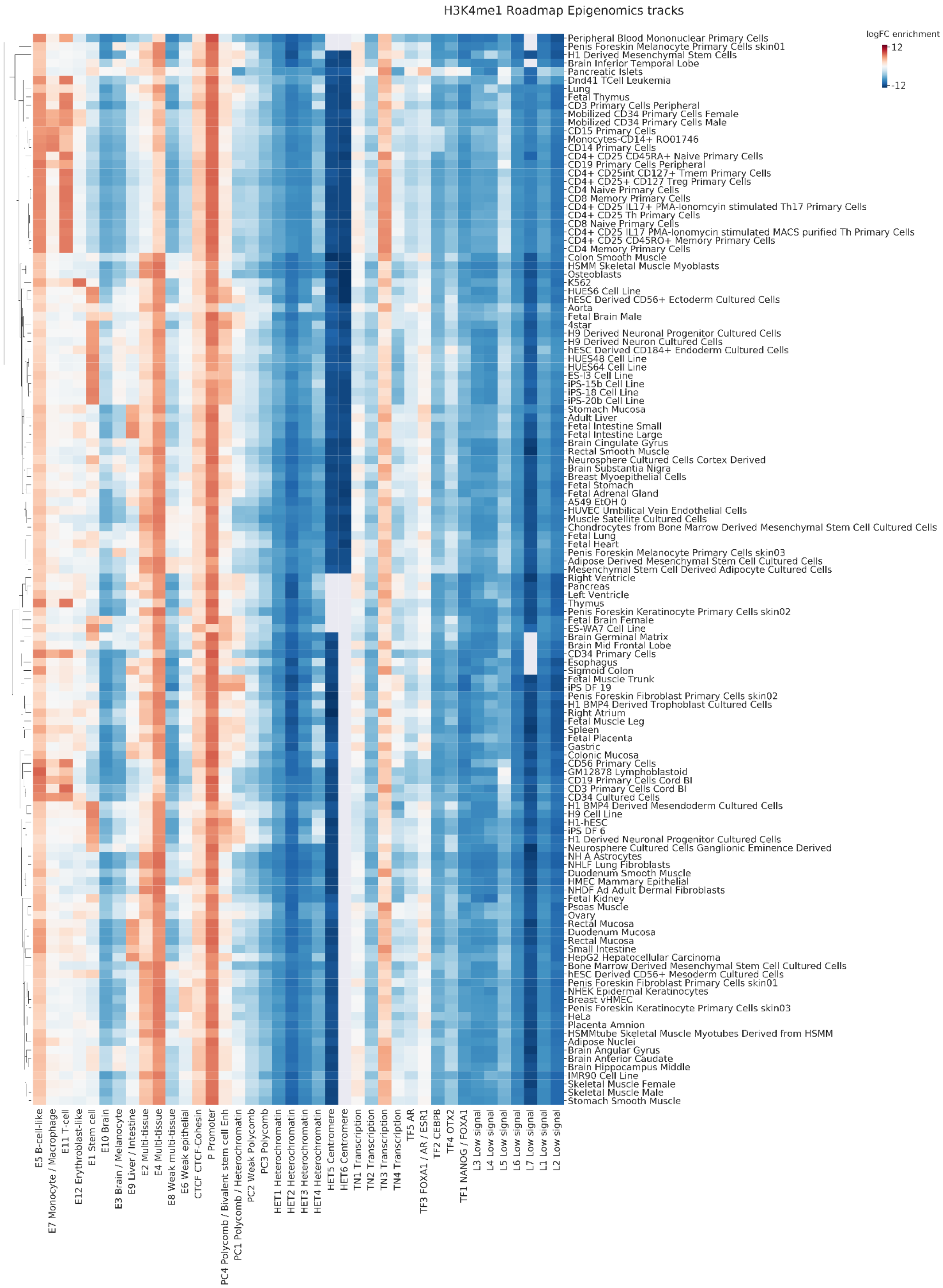

Supplementary Figure 8. Enrichment of tissue/cell type-specific H3K4me1 (enhancer mark) profiles in sequence classes. Log fold-change enrichment over genome-average background is shown in the heatmap. No overlap is indicated by the gray color in the heatmap.

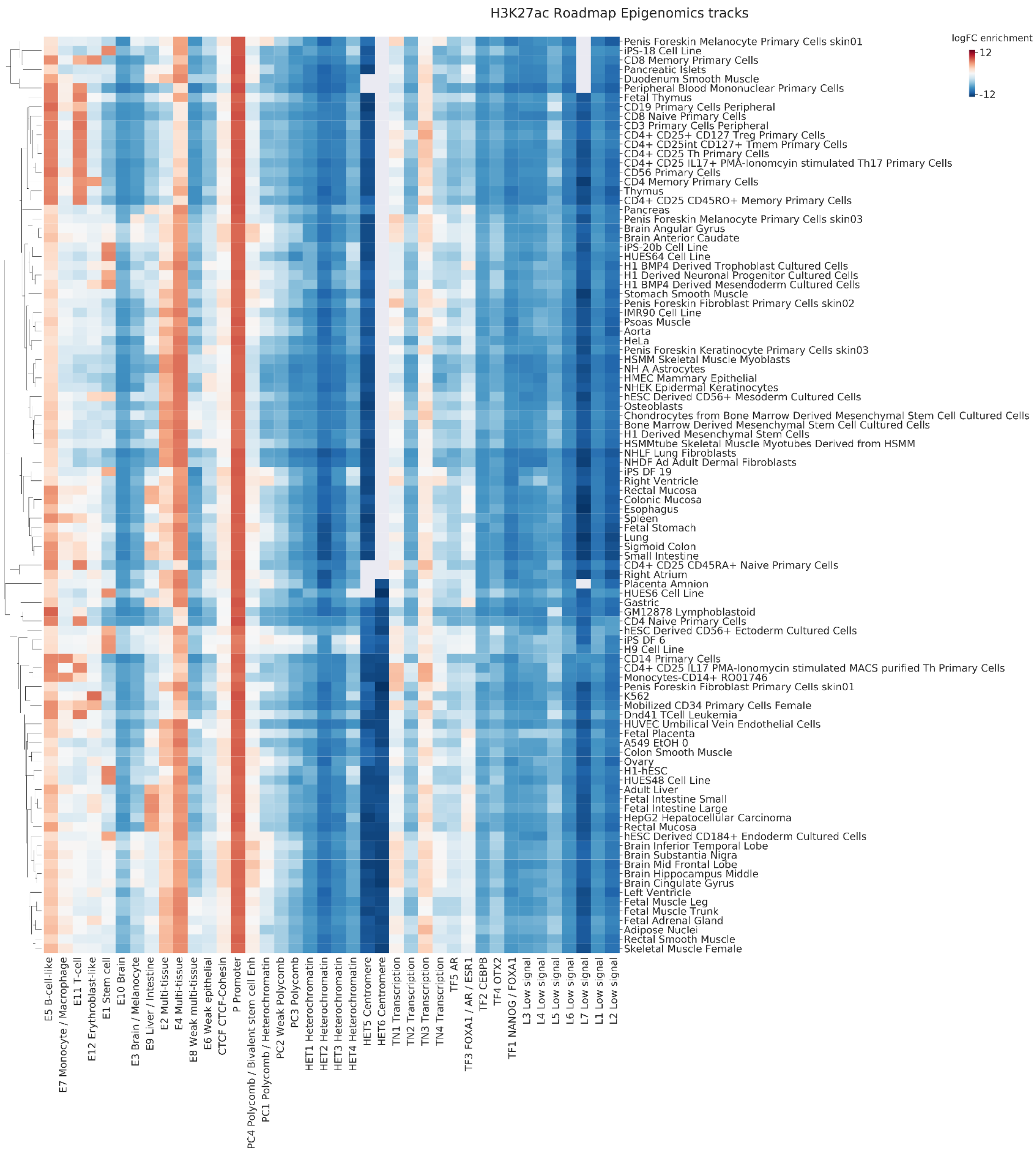

Supplementary Figure 9. Enrichment of tissue/cell type-specific H3K27ac (enhancer mark) profiles in sequence classes. Log fold-change enrichment over genome-average background is shown in the heatmap. No overlap is indicated by the gray color in the heatmap.

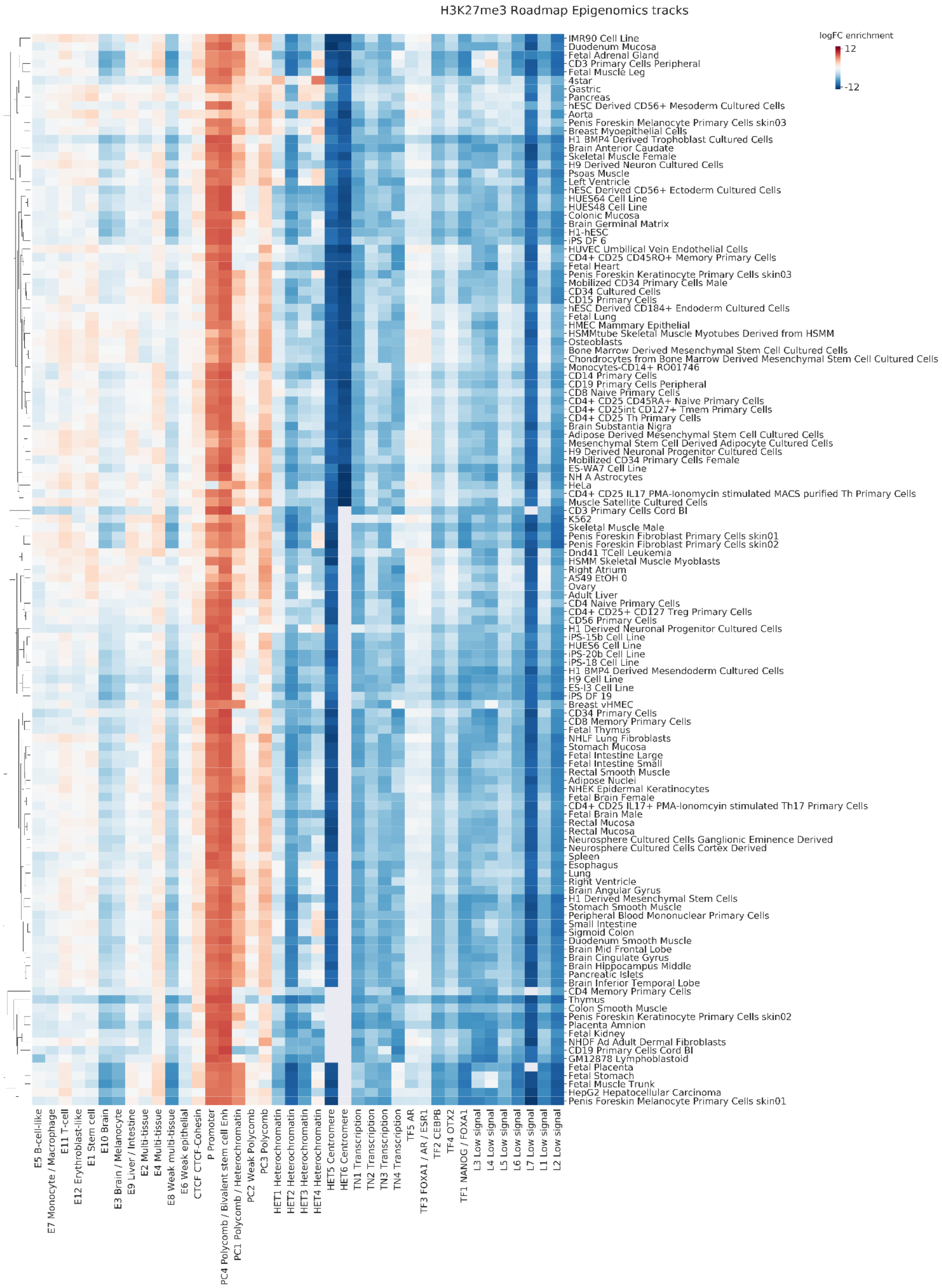

Supplementary Figure 10. Enrichment of tissue/cell type-specific H3K27me3 (Polycomb mark) profiles in sequence classes. Log fold-change enrichment over genome-average background is shown in the heatmap. No overlap is indicated by the gray color in the heatmap.

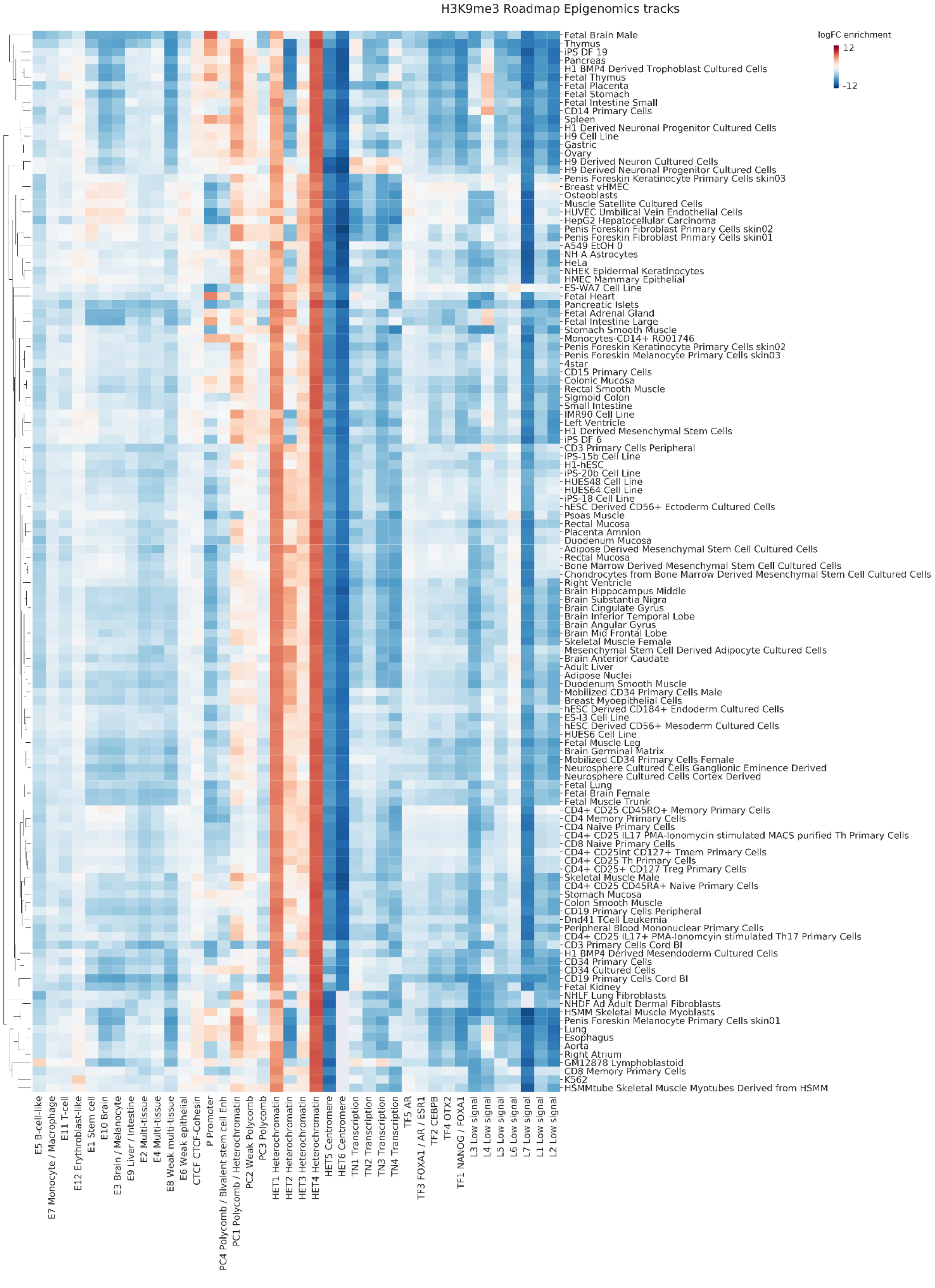

Supplementary Figure 11. Enrichment of tissue/cell type-specific H3K9me3 (heterochromatin mark) profiles in sequence classes. Log fold-change enrichment over genome-average background is shown in the heatmap. No overlap is indicated by the gray color in the heatmap.

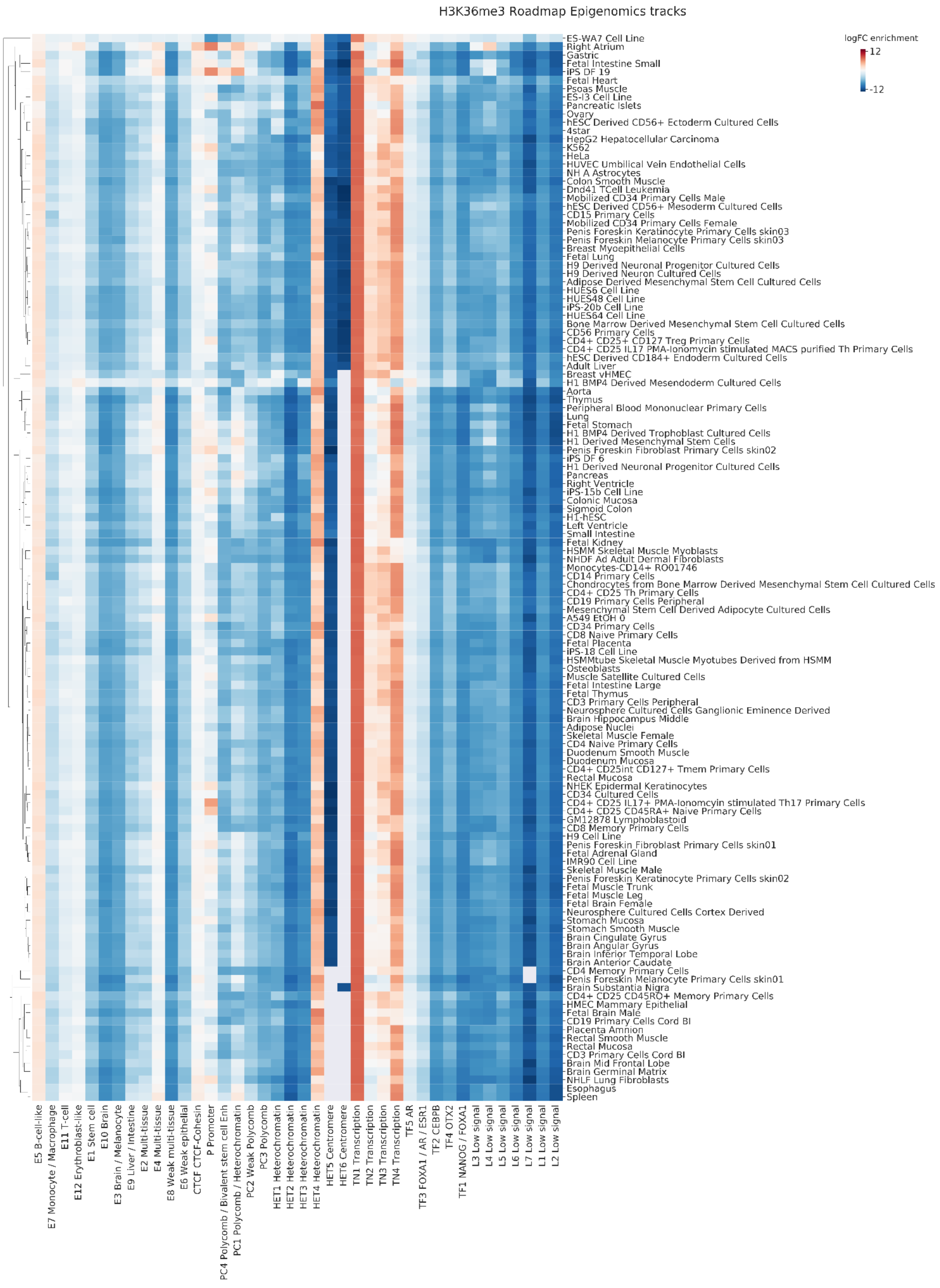

Supplementary Figure 12. Enrichment of tissue/cell type-specific H3K36me3 (transcription mark) profiles in sequence classes. Log fold-change enrichment over genome-average background is shown in the heatmap. No overlap is indicated by the gray color in the heatmap.

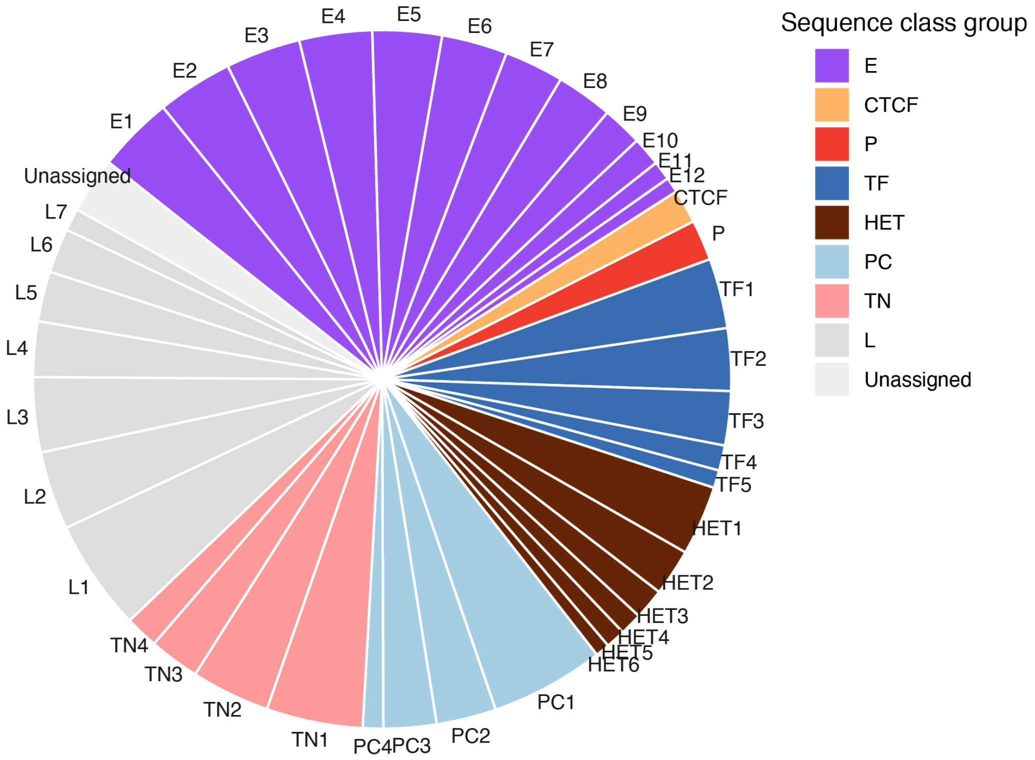

Supplementary Figure 13. Genome sequence proportion covered by each sequence class. The proportion of each sequence class is shown in the pie chart. Genome-wide sequence class assignments were based on Louvain clustering of Sei predictions of sequence tiling the genome with 100bp step size. The clusters unassigned to sequence classes due to the small size (below top 40 clusters) were categorized as “Unassigned”.

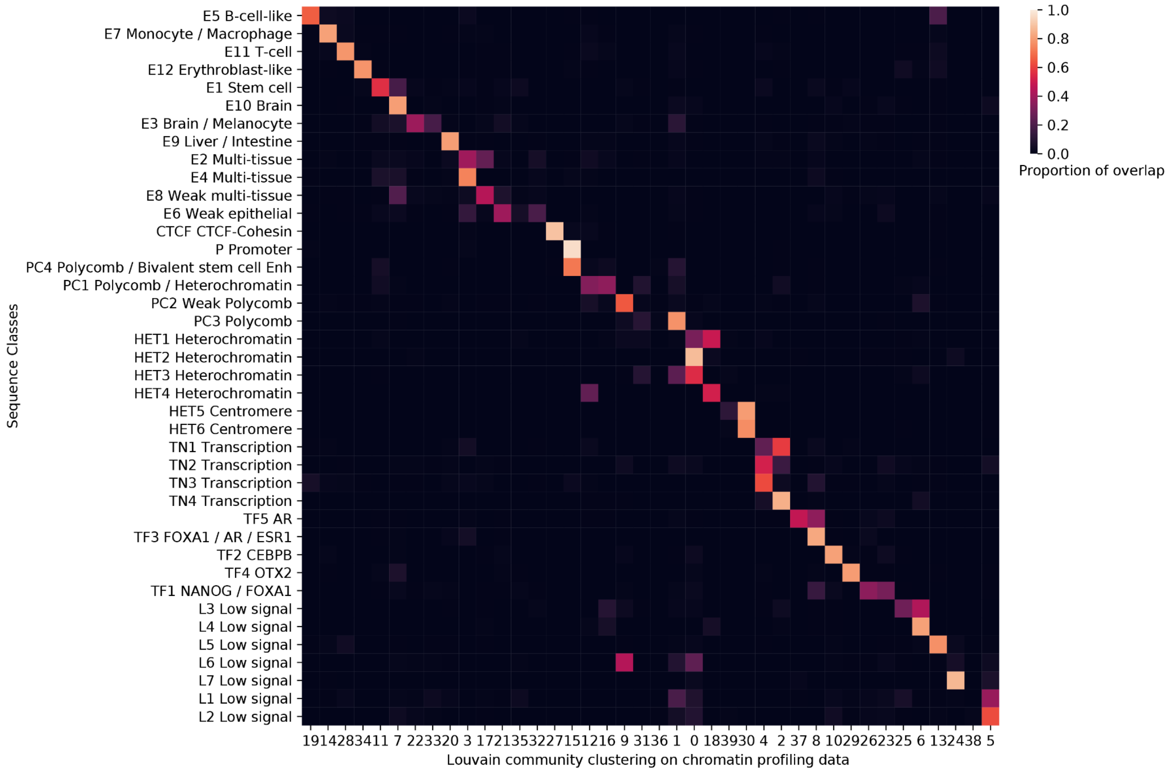

Supplementary Figure 14. Comparison of sequence classes and Louvain community clustering of the chromatin profiling data. For each sequence class, the proportion of overlap was computed between sequence classes and Louvain community clustering of the chromatin profiling data. The clustering is highly concordant with the current sequence class clusters.

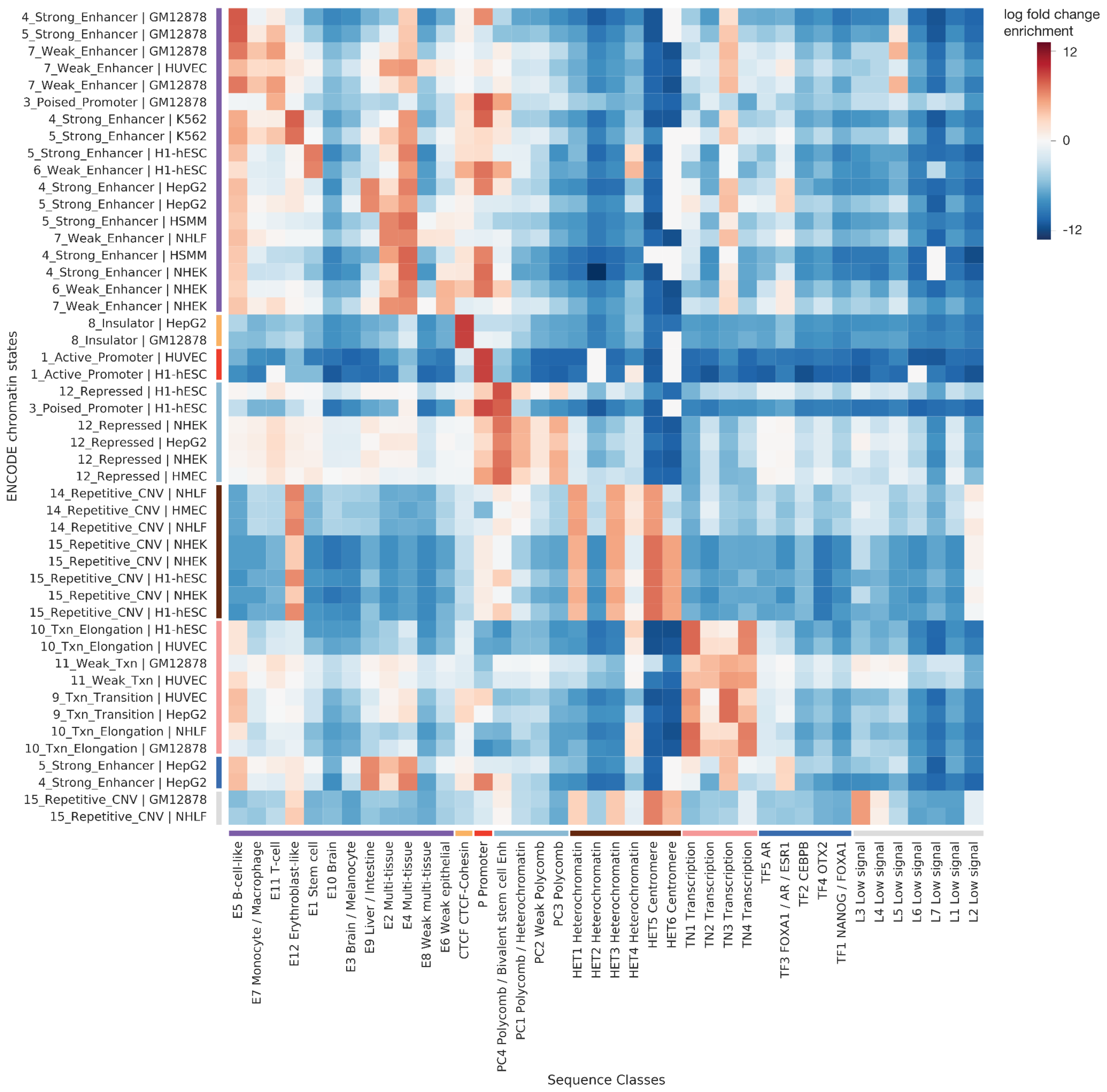

Supplementary Figure 15. Sequence-class-specific enrichment of ENCODE chromatin states. Log fold-change enrichment over genome-average background is shown in the heatmap. Top 2 chromatin states enriched were selected for each sequence class.

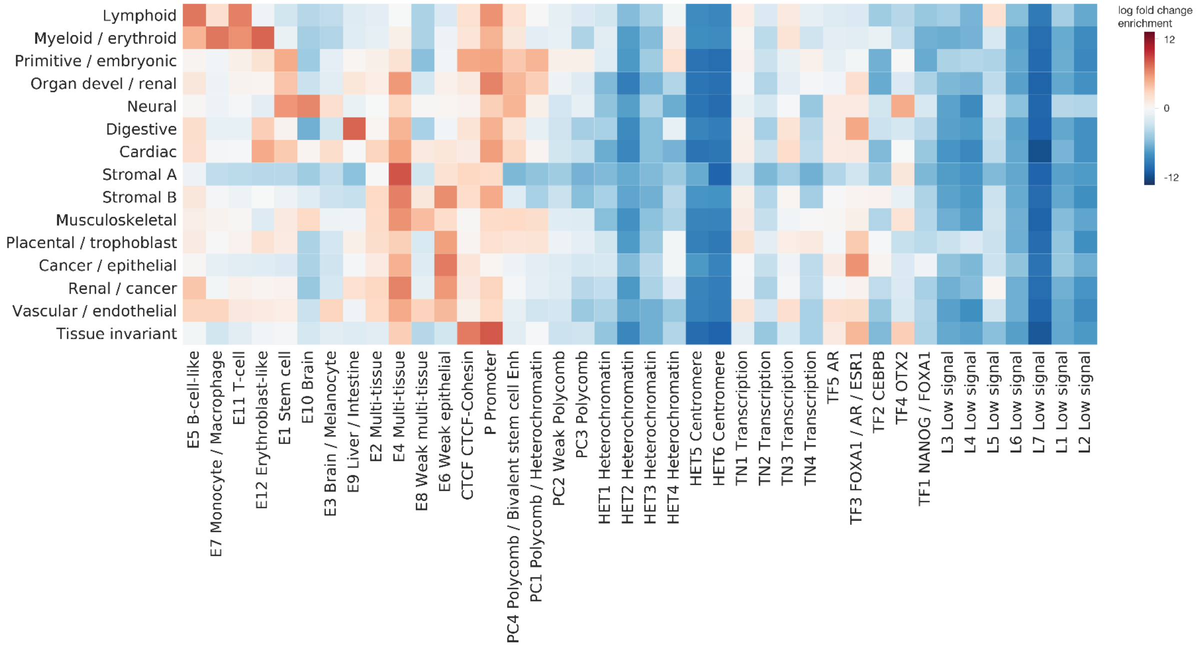

Supplementary Figure 16. Sequence-class-specific enrichment of tissue-specific DHS vocabulary [27]. Log fold-change enrichment over genome-average background is shown in the heatmap.

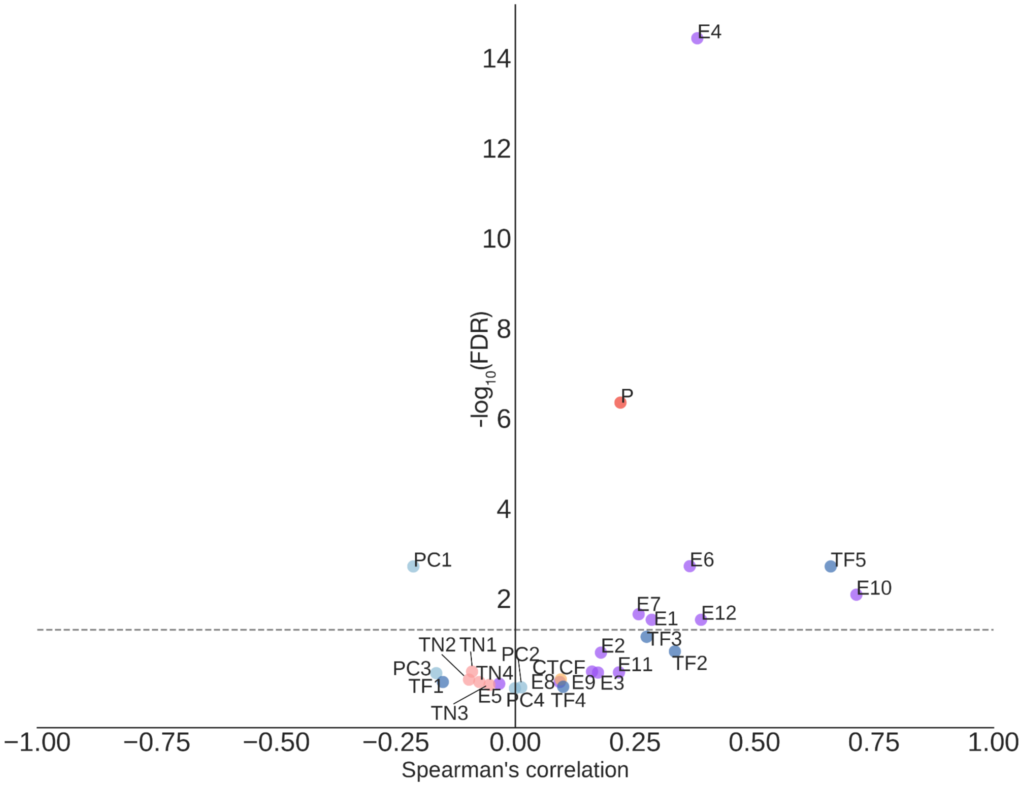

Supplementary Figure 17. Regulatory sequence-class-level variant effects for SNPs with PIP > 0.95 are predictive of directional GTEx variant gene expression effects. Variants assigned to sequence

classes based on the sequence class annotation for the reference genome. The x-axis shows Spearman correlations between the predicted sequence-class-level variant effects and the signed GTEx variant effect sizes (slopes) and the y-axis shows the corresponding log10 p-values. The dotted gray line denotes the Benjamini-Hochberg FDR < 0.05 threshold.

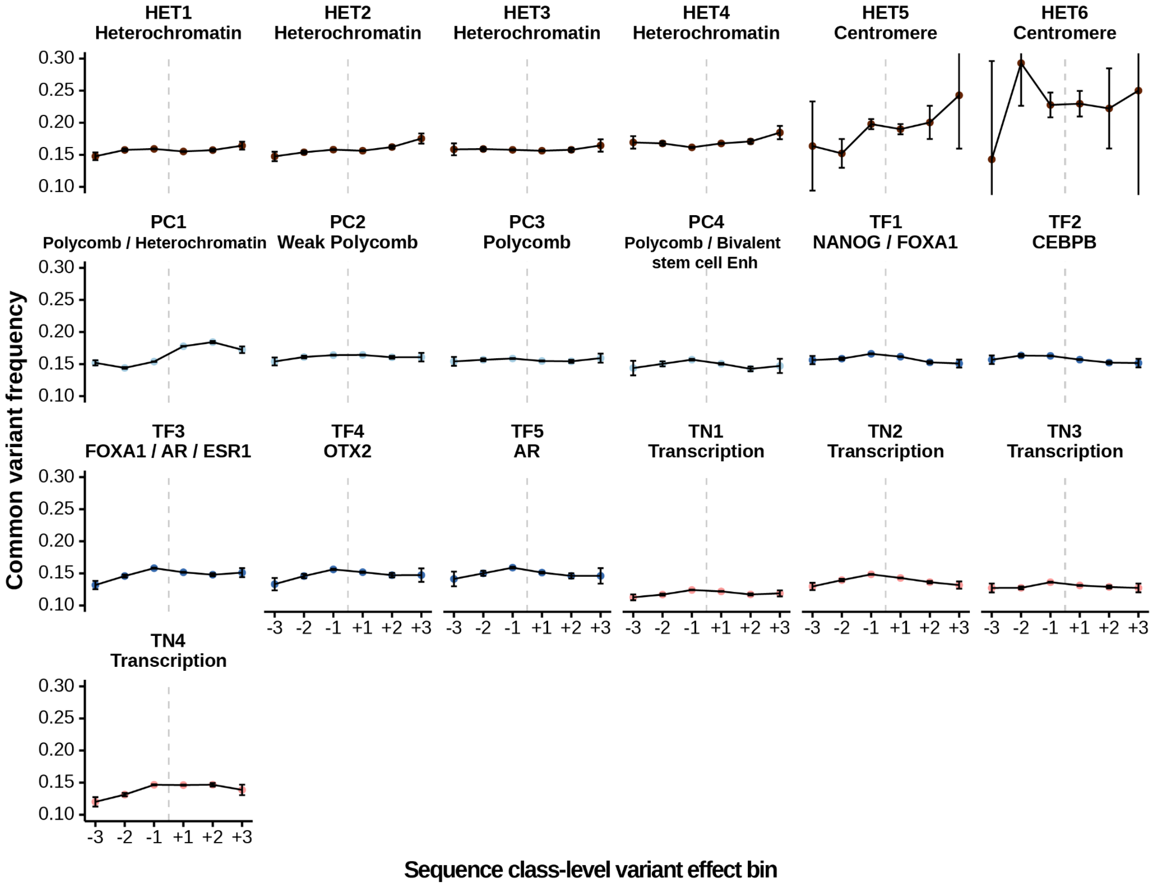

Supplementary Figure 18. Population allele frequency profiles for variants in heterochromatin, low signal, Polycomb, and transcription sequence classes. Comparison of common variant frequencies of 1000 Genomes variants (n=81,501,608) assigned to different sequence classes and variant effect bins. The common variant threshold is >0.01 allele frequency (n=12,803,919) across the 1000 Genomes population. Error bars show +/- 1 standard error (SE), and the center of error bars represents the mean. The sequence-class-level variant effects are assigned to 6 bins (+3: top 1% positive, +2: top 1-10% positive, +1, top 10-100% positive, -3: top 1% negative, -2: top 1-10% negative, -1, top 10-100% negative).

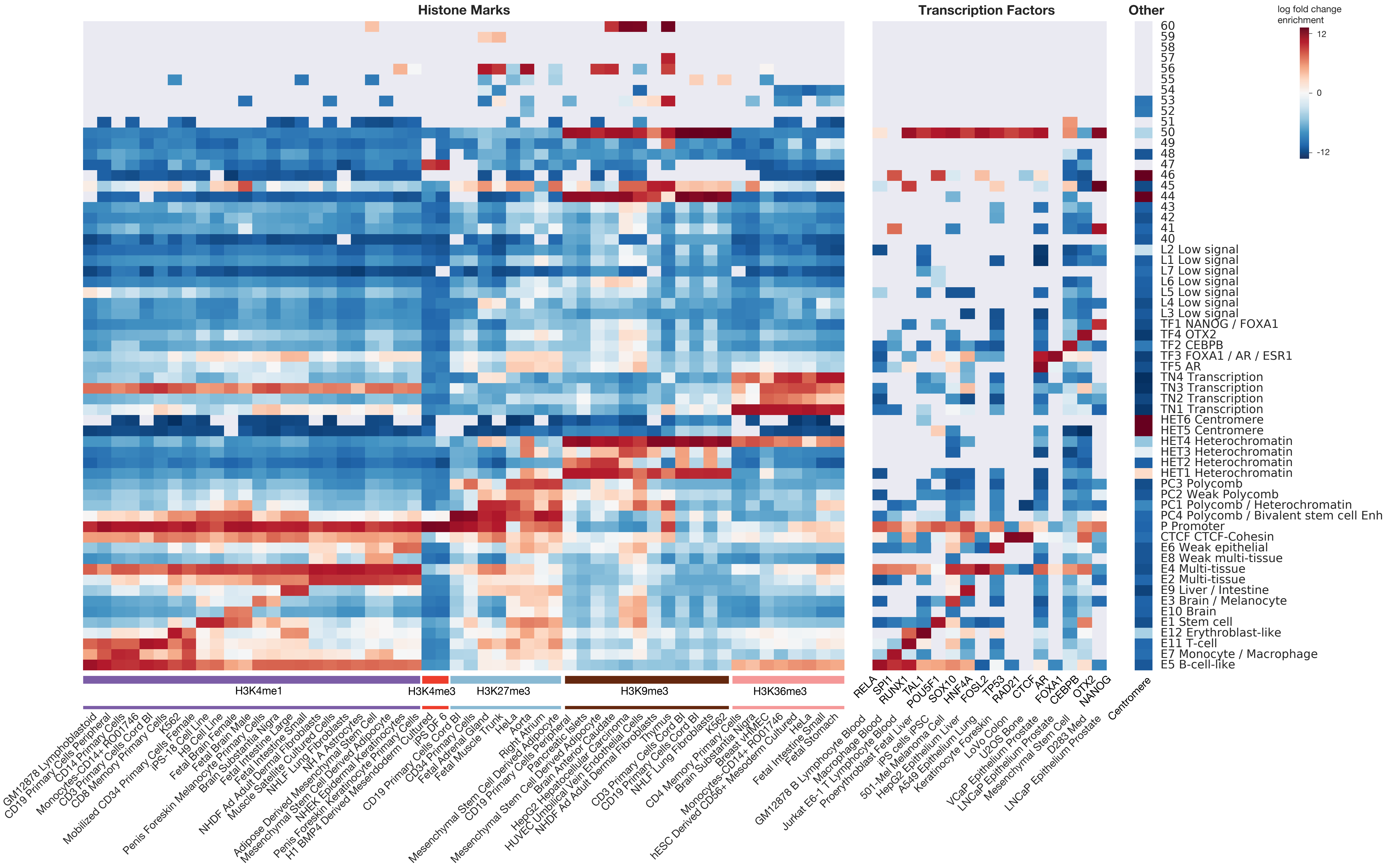

Supplementary Figure 20. Enrichment of histone marks, transcription factors, and repeat annotations for the full set of 61 clusters output by Louvain community clustering. Log fold-change enrichment over genome-average background is shown in the heatmap. No overlap is indicated by the gray color in the heatmap. Top 1-2 histone mark and TF annotation enrichments were selected for each sequence class.

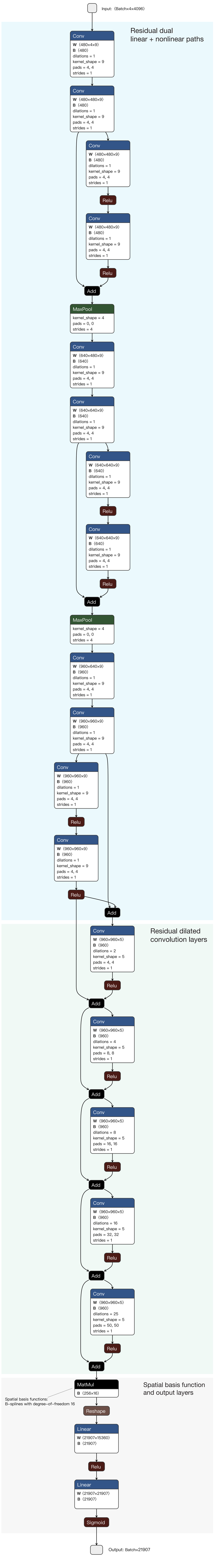

Supplementary Figure 21. Detailed Sei model architecture specification.
