# Supplementary material for: A sequence-based global map of regulatory activity for deciphering human genetics
Source: Nat Genet. 2022 Jul 11;54(7):940–9. doi: 10.1038/s41588-022-01102-2 (PMC9279145; doi:10.1038/s41588-022-01102-2)
Supplement: Supplementary file 4 — Top 25 enriched Cistrome Project chromatin profiles for each sequence class. [file 41588_2022_1102_MOESM4_ESM.pdf]

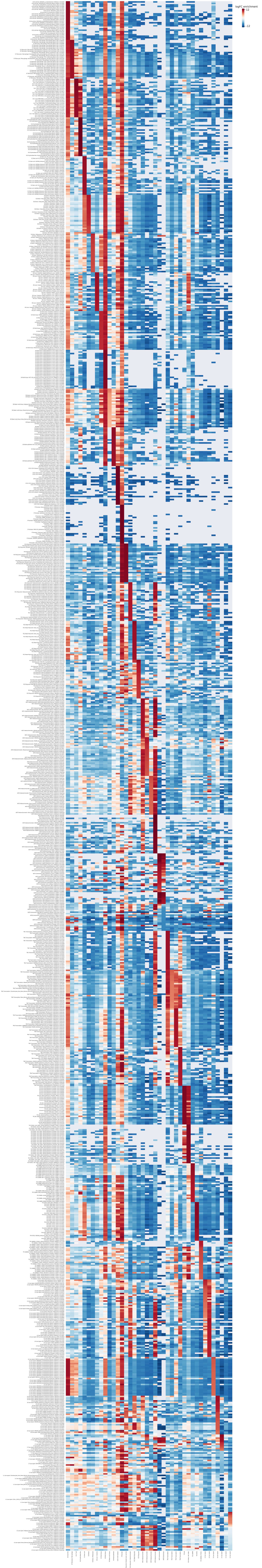

Supplementary Data. Top 25 enriched Cistrome Project chromatin profiles for each sequence class. Log fold-change enrichment over genome-average background is shown in the heatmap. No overlap is indicated by the gray color in the heatmap. We computed the enrichment for all 21,907 profiles predicted by Sei over 2 million random genomic positions. For each sequence class, the chromatin profiles are filtered to those having Benjamini-Hochberg corrected p-values (Fisher's exact test, two-sided) < 2.2e-16 selecting the top 25 profiles based on log fold-change enrichment.
